# Quantum biological tunnel junction for electron transfer imaging in live cells

**DOI:** 10.1038/s41467-019-11212-x

**Published:** 2019-07-19

**Authors:** Hongbao Xin, Wen Jing Sim, Bumseok Namgung, Yeonho Choi, Baojun Li, Luke P. Lee

**Affiliations:** 1 Institute of Nanophotonics, Jinan University, 511443 Guangzhou, China; 2 Berkeley Sensor and Actuator Centre, University of California at Berkeley, Berkeley, CA 94720 USA; 3 Department of Bioengineering, University of California at Berkeley, Berkeley, CA 94720 USA; 4 Biomedical Institute for Global Health Research and Technology, National University of Singapore, Singapore, 117599 Singapore; 5 Department of Bio-convergence Engineering, School of Biomedical Engineering, Korea University, Seoul, 02841 Republic of Korea; 6 Department of Electrical Engineering and Computer Sciences, University of California at Berkeley, Berkeley, CA 94720 USA; 7 Biophysics Graduate Program, University of California at Berkeley, Berkeley, CA 94720 USA; 8 Brigham and Women’s Hospital, Harvard Medical School, Boston, MA 02115 USA

**Keywords:** Nanoparticles, Imaging and sensing, Biosensors, Nanophotonics and plasmonics, Molecular imaging

## Abstract

Quantum biological electron transfer (ET) essentially involves in virtually all important biological processes such as photosynthesis, cellular respiration, DNA repair, cellular homeostasis, and cell death. However, there is no real-time imaging method to capture biological electron tunnelling in live cells to date. Here, we report a quantum biological electron tunnelling (QBET) junction and its application in real-time optical detection of QBET and the dynamics of ET in mitochondrial cytochrome *c* during cell life and death process. QBET junctions permit to see the behaviours of electron tunnelling through barrier molecules with different barrier widths. Using QBET spectroscopy, we optically capture real-time ET in cytochrome *c* redox dynamics during cellular apoptosis and necrosis in living cells. The non-invasive real-time QBET spectroscopic imaging of ET in live cell open a new era in life sciences and medicine by providing a way to capture spatiotemporal ET dynamics and to reveal the quantum biological mechanisms.

## Abstract (web summary)

Although quantum biological electron transfer is important in many biological processes, imaging of the events in live cells has remained challenging. Here, the authors demonstrate real-time optical detection of quantum biological electron tunnelling between nanoparticles and cytochrome c inside living cells.

## Introduction

As one of the simplest and the most fundamental chemical reaction, electron transfer (ET) reaction is of crucial importance in biology, chemistry, and almost every aspect of life. Since the pioneering theoretical work by Marcus more than 60 years ago^1^, efforts on understanding of ET in enzymes in biological reactions and biological systems have been made constantly^2–4^. ET in enzymes governs the basic biochemical reaction in the electron transport chain of two most important metabolic processes, i.e., photosynthesis^5^ and respiration^6^. It has also been proposed that ET plays crucial roles in many other biological processes in living systems, such as avian magnetoreception^7^, olfaction^8^ and vision^9^. In addition to the importance of intracellular ET, extracellular ET plays critical roles in many different biogeochemical applications. For example, ET from microbes to extracellular electron acceptors via microbial nanowires^10^ or secreted compounds such as quinones^11^ and flavins^12^ are important in organic matter degradation, subsurface bioremediation and nutrient cycling in soils and sediments. Although the importance of its role has long been highlighted, study of the quantum mechanical ET in biological systems was previously limited to mathematical modelling and theoretical conjecture^13^. Specific ET pathways encoded in protein structures determine the biological processes by providing low-lying electronic states via a superexchange-mediated mechanism through a tunnelling barrier^14^. Despite the great advancement in theoretical understanding of quantum biology recently^15^, experimental observation of quantum biological electron tunnelling has still been limited. Particularly, experimental visualisation of real-time quantum biological electron tunnelling (QBET) in biological systems is technically challenging.

In addition to the critical function of ET in living biological systems, understanding ET in enzymes is also very important in biofuel cells^16^, nanoreactors^17^ and biosensors^18^. Traditionally, electrochemical techniques have been adopted to characterise ET between the redox active sites of immobilised enzymes and the supporting electrodes^19^. The molecular ET in enzymes was detected previously via direct physical contact of the enzymes with an electrode using electric wiring or plugging enzymes into nanomaterials such as carbon nanotubes^20^ and gold nanoparticles (GNPs)^21^. These methods were applied for the measurement of ET in enzymes or enzyme assembles. Recent studies of molecular tunnel junctions (MTJs) have shown the capability of ET capture via quantum tunnelling through single molecules or self-assembled monolayers (SAMs)^22–24^. Typically, in the MTJs, two electrodes bridged by the molecules are utilised to capture the ET through the molecules via quantum tunnelling with an applied bias voltage.

On the other hand, plasmonic nanostructures with subnanometer-scale gaps provide a new platform for the investigation of quantum electron tunnelling^25–27^. When two plasmonic nanostructures are placed in close proximity with each other, plasmon response differs with the gap distance. If the gap is larger than a few nanometres (2–5 nm), strong plasmon coupling will promote local field enhancement at the gap^25^. However, as the gap distance becomes smaller, the system enters the quantum regime. The quantum nature of the electrons significantly alters the plasmon response. When the distance is less than a few nanometers (2–5 nm), the main quantum effect is nonlocal charge screening. As the gap further decreases to the threshold tunnel-distance at the sub-nanometer scale, the spill-out of plasmon-induced surface electrons can tunnel across the gap through a potential barrier at the optical resonant frequencies, and the quantum tunnelling is the main quantum effect in this regime.^25^ The threshold tunneling distance is found to be about 0.3~0.5 nm in vaccum^28^. However, the potential barrier can be lowered with the introduction of MTJs formed with linker molecules, and the tunnelling distance can be increased above subnanometer via through-bond tunneling^29^. Using electron energy-loss spectroscopy (EELS) method, Tan et al. recently described ET across the MTJs at length scales in the quantum gap (0.4–1.3 nm)^29^. All these previous methods are excellent demonstrations for validating ETs via tunnel junctions with an applied bias voltage. However, they are not suitable for visualising quantum biological ET in living molecules within live cells due to the wiring problem of electrodes on molecules^16–18,22–24^ or large scale of metallic substrates^27^, which are unfeasible for inserting into cells. Moreover, EELS-based quantum plasmonic detection method cannot be applied for live cells imaging due to the high-energy of the electron beam^29^.

Here, we report the first observation of QBET in living enzyme cytochrome *c* (Cyt *c*), which plays key functions in many metabolic processes as well as life and death decisions, such as respiration^30,31^, apoptosis activation^31^, the regulation of many diseases, such as diabetes^32^ and neurodegenerative disorders^33^. By using a biological tunnelling junction, real-time ET dynamics during Cyt *c* reduction process was observed at the molecular level via QBET spectroscopy. Finally, ET dynamics during apoptosis and necrosis in living cells were discerned by quantifying the oxidation and reduction of Cyt *c*. This study opens up a non-invasive and wireless optical means to transmit fundamental knowledge of QBET in biological systems via plasmonic optical antennas, with significant implications for future precision diagnostics and therapeutics.

## Results

### Quantum electron tunnelling behaviour in a tunnel junction

**Fig. 1:**
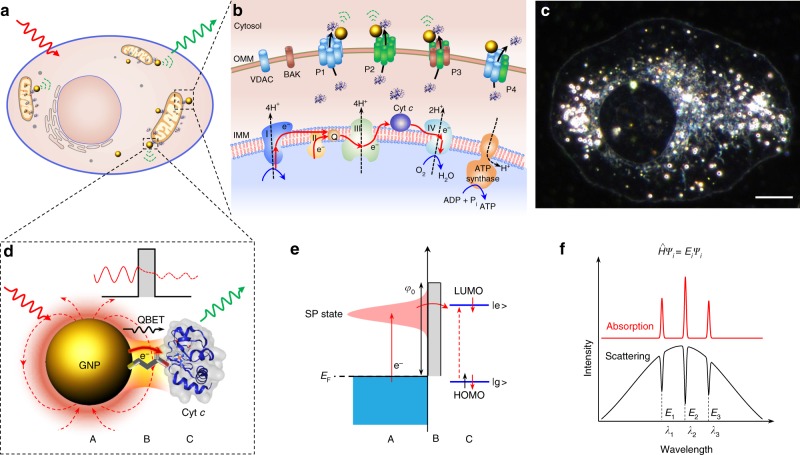
QBET imaging in a tunnel junction. **a** Schematic of GNPs used as optical antennas for intracellular QBET imaging in living cells. **b** Schematic illustration of GNPs for the detection of Cyt *c* released from mitochondria to cytosol. Cyt *c* is originally involved in the electron transport chain on the inner mitochondrial membrane (IMM), and transfers electrons between complexes III (Cyt *c* reductase) and IV (Cyt *c* oxidase, COX). After the formation of different pores or channels on outer mitochondrial membrane (OMM), Cyt *c* is released through these pores to cytosol. P1 to P4 represent pores based on oligomeric voltage-dependent anion channels (VDAC), BAX oligomer, BAX-BAK oligomer, and VDAC-BAX oligomer, respectively. BAX and BAK are two B-cell lymphoma protein-2 (BCL2) effector proteins. **c** A representative dark-field image of a living cell with GNP uptake. Scale bar: 10 µm. **d** Schematic of QBET in A/B/C tunnel junction. Single GNP acts as an Au plasmonic optical antenna (A) with light irradiation. Surface electrons collectively oscillate at the resonant light frequency. Electrons tunnel through the potential barrier via linker molecule (barrier molecular, B) to Cyt *c* (C). **e** Schematic illustration of electron tunnelling in the A/B/C tunnel junction. Electron is excited from Fermi level (*E*_F_) to a surface plasmon (SP) state, and tunnels through the potential barrier via the barrier molecule to Cyt *c*. The molecule is excited to the excited state (|e > ) after the electron tunnelling and transfer. |e > : excited state, |g > : ground state, LUMO: lowest unoccupied molecular orbital, HOMO: highest occupied molecular orbital. **f** Scattering spectra with quantised dips for QBET imaging. QBET results in quantised dips in the scattering spectra of GNP, which matches the frequencies of electronic transitions of Cyt *c* (absorption peaks). These dips correspond to the quantised eigenvalues *E*_*i*_ at the electron state *ψ*_i_ in the tunnelling process. The cartoons for Cyt *c* molecules in **a**, **b** and **d** were created from the RSCB protein data bank^54^

Plasmonic GNPs serve as optical antennas which enable real-time spectroscopic molecular imaging in living cells^34^. These optical antennas also enable molecular ET imaging of enzymes and the transmitting of QBET dynamics in live cells (Fig. 1a). We took Cyt *c* as an example of enzymes for the capture of ET. Cyt *c* is functional involved in ET within the electron transport chain in mitochondrial^35^ (Fig. 1b), and serves as a key participant in cell death control through apoptosis activation (see Supplementary Note 1 for more details). GNPs near the outer mitochondrial membrane act as nanoplasmonic antenna and are capable of capturing the released Cyt *c* from the mitochondria in living cells (Fig. 1b). Figure 1c shows a dark-field image of an intact living cell with GNP (50 nm, green) uptake, which we will discuss in detail for QBET imaging using these GNP-based optical antennas. Organelles inside the cell as well as their movement can be clearly seen (Supplementary Fig. 1 and Supplementary Movie 1). Cellular organelles show broad scattering spectra in the visible range, which is distinct from the spectra of GNPs with plasmonic peaks (Supplementary Fig. 1c).

For the visualisation of QBET in living cells, we capture electron tunnelling across an A/B/C tunnel junction (Fig. 1d). Cyt *c* (C) is conjugated with a GNP (Au, A) via linker molecules (barrier molecule, B), which forms a quantum tunnel junction with a potential barrier. The GNP serves as an optical antenna with wavelength-specific surface plasmon (SP) resonance with strong light scattering when excited with white light irradiation. Free conduction electrons are elevated from Fermi level (*E*_F_) to a higher energy level (SP state), and collectively oscillate at the GNP surface at the resonant light frequency. This system permits the plasmon resonance energy transfer (PRET) from the GNP to the conjugated Cyt *c* molecules. When the gap in the tunnel junction enters the quantum regime with a subnanometer distance, the spill-out of plasmon-induced surface electrons can tunnel across the gap at optical resonant frequencies^25^, and quantum electron tunnelling and transfer will coexist with PRET, playing key function in the modulation of the spectrum. Due to the linker molecules in the junction, the tunnel barrier height in the gap between the GNP and Cyt *c* is lower than that in vacuum without linker molecules, which facilitates electron tunnelling^29^, allowing ET from GNP to Cyt *c* (Fig. 1e). The height of potential barrier (*φ*_0_) is the offset between the Fermi level of the GNP and the lowest unoccupied molecular orbital (LUMO) of the linker molecule^29^. The width of tunnelling barrier is the length of the linker molecule. The molecule is excited to the excited state (|e > ) after the electron tunnelling and transfer. As a result of the electron tunnelling, we observe quantised dips located at Cyt *c* absorption peaks in the scattering spectrum of the GNP (Fig. 1f) (see the discussion in Supplementary Note 2), enabling optical capture of QBET in the tunnel junction. The details of the underlying mechanism are, however, not yet fully understood.

**Fig. 2:**
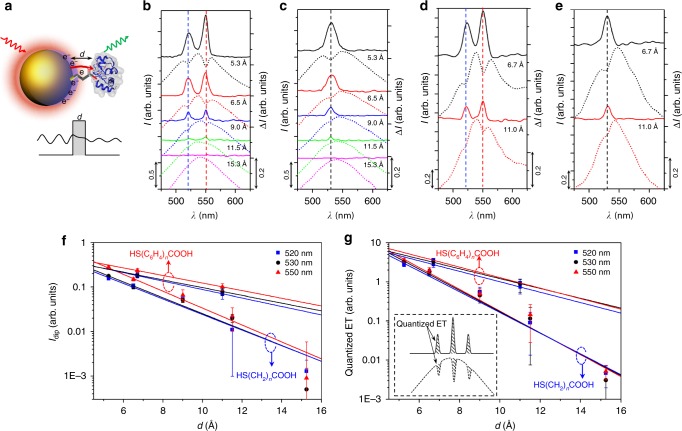
Tunnelling behaviour with different barrier width. **a** Schematic of electron tunnelling in A/B/C tunnel junction with variable barrier width *d*. The cartoon for Cyt *c* molecule was created from the RSCB protein data bank^54^. **b**–**e** Scattering spectra and spectra  difference for QBET imaging in (**b**, **d**) A/B/C (Red.) and (**c**, **e**) A/B/C (Ox.) tunnel junction with (**a**, **b**) HS(CH_2_)_*n*_COOH and (**c**, **d**) HS(C_6_H_4_)_*n*_COOH of different barrier widths as linker molecules. The quantised peaks were obtained from the difference of scattering spectra between the GNP-Cyt *c* conjugation and the linker molecule modified GNP without Cyt *c*. Dashed curves are captured scattering spectra (linked to left axis) of GNP-Cyt *c* conjugation, and solid curves are the corresponding spectra difference (linked to right axis). Blue and red dashed lines in **b**, **d** indicate the quantised dips (peaks) at 520 and 550 nm, respectively, while the black dashed lines in **c**, **e** indicate the dips (peaks) at 530 nm. **f** Dip depth (*I*_dip_) as a function of barrier width with exponential fitting. The red dashed circle with an upwards arrow and blue dashed circle with a downwards arrow indicate the two groups of data are for HS(C_6_H_4_)_*n*_COOH and HS(CH_2_)_*n*_COOH, respectively. **g** Quantised ET as a function of barrier width, experimental data are fitted using the quantum tunnelling equation. Inset shows the schematic of accumulated quantised ET from the integral of the dips in the scattering spectra. Error bars represent standard deviation

To quantify electron tunnelling across the A/B/C tunnel junction with different tunnelling barrier widths *d* (Fig. 2a), we used different lengths of linker molecules. Carboxyl-terminated alkanethiol HS(CH_2_)_*n*_COOH (*n* = 2, 3, 5, 7 and 10) (Supplementary Table 1), with corresponding lengths of 5.3, 6.5, 9.0, 11.5 and 15.3 Å, were first used to conjugate the GNP and Cyt *c* (see Supplementary Note 3 for more discussion on the conjugation). We used spherical GNPs with a diameter of 50 nm, which have a plasmonic scattering peak of around 530–540 nm. This plasmonic peak shows a good match and overlap with the absorption peaks of Cyt *c* in both reduced (Red.) and oxidised (Ox.) form (Supplementary Fig. 2). The plasmonic resonance enables plasmon-induced electrons tunnelling from GNP to Cyt *c* at the resonant optical frequencies, and the matched frequencies with absorption peak of Cyt *c* induce the electronic excitation of the Cyt *c* at the specific wavelength. Although the extinction coefficient of a single GNP is a few orders of magnitude higher than that of a single Cyt *c* molecule (Supplementary Fig. 3), the QBET effect captured in this study is not a one-to-one event between a GNP and a Cyt *c*, and the quantised discrete dip is the cumulative results of continuous quantum electron tunnelling between a GNP and many conjugated Cyt *c* molecules. We also observed that the effective concentration of Cyt *c* should be higher than 1 μM to form A/B/C junctions for successful detection of QBET signal.

First, we demonstrated tunnelling behaviour using Cyt *c* (Red.). Decreasing dip depths at both 520 and 550 nm, corresponding to the absorption peaks of Cyt *c* (Red.), were captured as the barrier width was increased (Fig. 2b). An effective tunnelling distance of 11.5 Å was obtained. No discernible dips were found with a barrier width of 15.3 Å, indicating that the electron was not able to tunnel through such a long barrier width. Similar to the reduction case, the electron was not able to tunnel through the barrier width of 15.3 Å (no dip captured) in the A/B/C (Ox.) tunnel junction (Fig. 2c). The dip depths with different barrier widths for A/B/C (Red.) (520 and 550 nm) and A/B/C (Ox.) (530 nm) junctions are shown in Fig. 2f. Experimental data were exponentially fitted, giving a dip decay constant of 0.41, 0.42, and 0.44 Å^−1^ at 520, 530 and 550 nm, respectively. In the A/B/C tunnel junction, the captured ET from the quantised dips of the scattering spectra is resulted from the accumulation of a large number of electrons tunnelling, and can be estimated by the area integral of the dips (Fig. 2g). The exponentially fitted values of tunnelling decay rate (*β*) for the electrons corresponding to the wavelengths of 520, 530 and 550 nm are 0.61, 0.63 and 0.64 Å^−1^, respectively, with an average tunnelling decay rate of 0.63 Å^−1^. This value is consistent with those measured from larger-area molecular junctions by Akkerman et al.^36^, who report *β* values within 0.57–0.66 Å^−1^ for SAMs of alkanethiols sandwiched within a gold electrode and a conductive polymer electrode. These values are smaller than the theoretical values of 1.46, 1.48 and 1.51 Å^−1^ based on a single electron tunnelling through the barrier (Supplementary Fig. 4). A major factor inducing this difference between the theoretical and experimental values is the accumulation of a large number of electrons in the experiments due to surface plasmon excitation. This accumulation of electrons can induce a significant difference in the tunnelling behaviour from a single electron. Another factor may be the hopping of electrons in the junction, which significantly decreases the average height of the barrier, eventually increasing the electron transfer rate. The tunnelling can also involve either intermolecular tunnelling though the carbon backbone of the molecule or intramolecular charge transport that involve multiple molecules^37^. It should be noted that Cyt *c* is macromolecular protein composed of 104 amino acid residues, and the iron ion is in the centre of the heme *c* structure inside the protein, the transferred electron can be transiently located at either the protein surface or any amino acid residues, and also can be released into the surrounding media. In either case, the electron cannot reduce the Cyt *c* (Ox.) into Cyt *c* (Red.). That is why we did not capture the reduction of Cyt *c* (Ox.) into Cyt *c* (Red.) in the electron transfer process.

The dips are measured in a discrete manner at specific molecular resonance wavelengths (molecular electronic states), and the depths of the measured dips differ from linker to linker molecules with subnanometer scale distance-dependent modulation. This is why we call the dips “quantised”, and these phenomena indicate that quantum electron tunnelling and transfer through the potential barrier plays key function during the dip generation. The quantised dips coincide precisely at frequencies that match electronic excitation frequencies of Cyt *c* (absorptions peaks). We suggest that, electron transfer from GNP to Cyt *c* with subsequent electronic excitation of Cyt *c* results in quantised energy loss in GNP governed by the general time-independent Schrödinger equation, which results in the dip generation in the scattering spectrum of the GNP. There may be other quantum mechanisms underlying the dip generation that are not fully understood yet. For general energy transfer processes, such as plasmon-induced resonance energy transfer^38^ and fluorescence resonance energy transfer^39^, the effective distance can go up to 10 nm, which is much larger than the 1.15 nm reported in our system. Previous study shows that the new peak of the spectrum will be red shifted with respect to the original one due to the localised surface plasmon resonance (LSPR) effect^40^. Indeed, we have also observed small shift (<1 nm) in the scattering spectrum of the GNP-Cyt *c* conjugation after the binding of Cyt *c* on the GNP surface (Supplementary Fig. 5). The theory of LSPR also explains well localised changes on plasmonic particle due to the changes of permittivity of surrounding media with the binding of biomolecules on particle surface^41^. However, the LSPR theory does not allow to explain the captured quantised dips in the spectrum at specific electronic states of molecules in the conjugation. Even though original plasmonic spectrum is slightly red-shifted, the quantised dips in the spectrum is a dominant signal in our system, which is much more discernible than the spectrum redshift. This is why we are proposing a new mechanism that was previously not discussed for quantum tunnelling in between plasmonic particle and biomolecule. This tunnelling method provides a new way to capture the quantum electron transfer in between nanoplasmonics and biomolecules, which is not possible to observe specific electronic states of molecules using spectrum shift method

In order to further demonstrate that electron transfer via quantum tunnelling through the potential barrier in the tunnel junctions plays key function in the generation of the quantised dips, we performed additional experiments with aromatic linker molecules, HS(C_6_H_4_)_*n*_COOH (*n* = 1, 4-Mercaptobenzoic acid, 4MBA; *n* = 2, 4′-mercapto-biphenyl-4-carboxylic acid, 4MB4CA) (Supplementary Table 1), to form tunnel junctions with different potential barrier between the GNP and Cyt *c*. The barrier height defined by the offset between the Fermi level (−5.5 eV) and the LUMO of the linker molecules are calculated to be ~4.93 and 3.65 eV for tunnel junctions formed with the representative linker molecules of MPA (HS(CH_2_)_2_COOH, 3-mercaptopropionic acid) and 4MBA, respectively (Supplementary Fig. 6). Due to the *π*-electron system in the aromatic phenyl rings, the aromatic molecules HS(C_6_H_4_)_*n*_COOH have smaller HOMO-LUMO gap than the carboxyl-terminated alkanethiol HS(CH_2_)_*n*_COOH, resulting in smaller potential barrier height in the tunnel junctions. Consequently, the junctions formed by aromatic linker molecules (4MBA and 4MB4CA) show a higher tunnelling efficiency with greater quantised dip depth in the scattering spectra than that of alkane linkers with similar molecular length (Figs. 2d, e). The dip depths at the wavelengths of 520, 530, and 550 nm with gap distances formed by different linker molecules is also shown in Fig. 2f, while the accumulated quantised ET is shown in Fig. 2g. The average tunnelling decay rate *β* for the linker molecules of HS(C_6_H_4_)_*n*_COOH for the electrons at the three resonant wavelengths is measured to be 0.31 Å^−1^ (Fig. 2g). This value is smaller than the average of 0.63 Å^−1^ for HS(CH_2_)_*n*_COOH due to the smaller potential barrier height of HS(C_6_H_4_)_*n*_COOH. This barrier height-dependent variation in the tunnelling behaviour further supports that the electron tunnelling through a barrier formed by the liker molecules exists and plays critical roles in the quantised dip generation, and QBET in this system can be captured from these dips using this tunnel junction. To keep the experimental condition consistent, we used MPA as a linker molecule and the barrier molecule to form the tunnel junction in the following experiments.

Although we observed similar phenomenon of quantised energy states (dips) within the broad scattering spectra of GNP-Cyt *c* conjugations before^42^, and explained that the dips were due to the PRET only from GNP to Cyt *c* based on dipole-dipole interaction without considering electron transfer. At that time, we were not able to discern that quantum electron tunnelling and transfer during the dip generation process due to the limited knowledge and limited experimental data (i.e., no tunnelling junction experiments). The results shown in Fig. 2 demonstrate that the generated quantised dips are highly dependent on the tunnel barrier width within the subnanometer scale, and these dips also significantly differs with potential barrier height. These results support that quantum electron tunnelling through the barrier formed by the linker molecules exists and plays critical roles in the dip generation. This new discovery is very important for the optical imaging of quantum electron tunnelling using plasmonic optical antennas.

### Real-time QBET imaging during Cyt *c* reduction

**Fig. 3:**
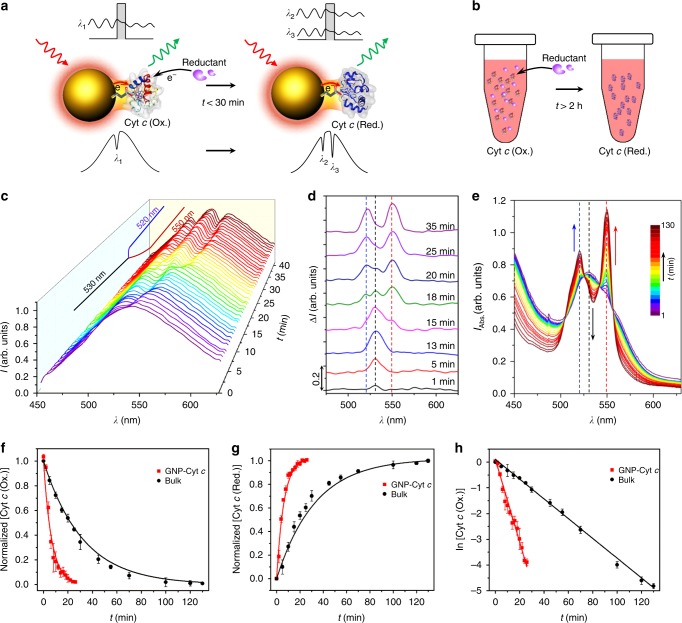
Real-time QBET imaging during Cyt *c* reduction. **a** Schematic for QBET imaging during Cyt *c* reduction in A/B/C tunnel junction, with a total reaction time of less than 30 min. With reductant added, Cyt *c* (Ox.) is reduced to Cyt *c* (Red.), and the electron tunnelling through the barrier changes from *λ*_1_ (530 nm) to *λ*_2_ (520 nm) and *λ*_3_ (550 nm). **b** Schematic for Cyt *c* reduction in bulk solution with a total reaction time of larger than 2 h. **c** Real-time scattering spectra of GNP-Cyt *c* conjugation in the whole Cyt *c* (Ox.) conjugating and reduction process, QBET in the reduction process is captured from the spectra dip changes which were splitted from 530 nm to 520 and 550 nm. **d** Representative scattering spectra difference (compared with the GNP without Cyt *c* at *t* = 0) in the reduction process showing the dip changes at different time. **e** Absorption spectra of Cyt *c* bulk solution during the reduction process. The absorption peak of 530 nm (dashed black line) for Cyt *c* (Ox.) is gradually decreased, while the peaks of 520 (dashed blue line) and 550 nm (dashed red line) for Cyt *c* (Red.) are gradually increased. **f** Normalised Cyt *c* (Ox.) concentration during the reduction process in both GNP-Cyt *c* conjunction and bulk solution. **g** Normalised Cyt *c* (Red.) concentration during the reduction process. **h** Plot of ln [Cyt *c*] in the reduction process with linear fit. The cartoons for Cyt *c* molecules in **a** and **b** were created from the RSCB protein data bank^54^. Error bars represent standard deviation

In living cells, Cyt *c* is capable of undergoing reduction and oxidation in the presence of Cyt *c* reductase and oxidase (COX) in inner mitochondrial membrane, respectively. In the cytosol, the pro-apoptotic activity of Cyt *c* is critically influenced by its redox state, regulated by the cytosolic environment^43^. Using the tunnel junction, we were able to capture the real-time ET in Cyt *c* during reduction via the QBET spectroscopy. The electron tunnelling at *λ*_1_ (~530 nm) in the junction was changed to *λ*_2_ (~520 nm) and *λ*_3_ (~550 nm) after reduction (Fig. 3a). Real-time QBET during Cyt *c* reduction was optically captured from the change of dip locations (*λ*_1_ to *λ*_2_ and *λ*_3_) with monitoring of a specific GNP. Cyt *c* (Ox.) molecules were first conjugated to the GNP via linker molecules. The captured dips in the scattering spectra were located at *λ*_1_ = 530 ± 2 nm (Fig. 3c, d), as a result of QBET in the A/B/C (Ox.) tunnel junction. At around *t* = 13 min, the dithionite anion (S_2_O_4_^2−^) approached the GNP, and the conjugated Cyt *c* (Ox.) started to reduce to Cyt *c* (Red.). Due to the QBET at *λ*_2_ and *λ*_3_ in the A/B/C (Red.) tunnel junction, a single dip began to shift and split into two dips at *λ*_2_ = 520 ± 2 nm and *λ*_3_ = 550 ± 2 nm during the reduction process (Fig. 3c, d). The whole reduction process took <30 min in the GNP-Cyt *c* system (Supplementary Fig. 7).

Next, the reaction in the GNP-Cyt *c* system with tunnel junction for QBET was compared to Cyt *c* reduction in bulk solution (Fig. 3b). Fig. 3e shows the reduction dynamics in bulk solution, while Fig. 3f–h shows the comparison with the reduction in GNP-Cyt *c* system (see the detailed description in Supplementary Note 4). From the respective exponential and linear fit in Fig. 3f, h, we get an average reaction rate constant *k* of 0.16 and 0.035 min^−1^ for the GNP-Cyt *c* and the bulk solution, respectively. These values indicate that the reaction in the plasmonic GNP-Cyt *c* system is ~4.6 times faster than that in the bulk solution. This enhancement implies that the QBET in A/B/C tunnel junction acts as a catalyst for the reaction. This catalytic effect is probably due to the ET and its related energy transfer from plasmonic GNP to Cyt *c*, which in turn decreased the activation energy of the reaction^44^.

### Real-time intracellular QBET imaging during cell death process

The real-time QBET imaging capability of A/B/C junction spectroscopy motivated us to capture intracellular ET of Cyt *c* under different cellular stimuli. This intracellular ET imaging is critical for better understanding of ET in governing cell death and provides new opportunities for precision intracellular molecular diagnostics and therapeutics. Cell death occurs via two different forms: apoptosis, a regulated natural process to maintain a balance of cellular multiplication, which plays a key role in maintain the balance of cell types and numbers^45^ as well as the regulation of disease such as neurodegenerative diseases^46^; and necrosis, a pathological process that occurs when cells are exposed to extreme conditions that are very different from the homeostatic condition. Although apoptosis and necrosis are morphologically and biochemically distinct, these cell death processes occur simultaneously in cells or tissues subjected to the same drug treatment^47^. The difference between apoptosis and necrosis is mainly inflicted by genes and metabolic states of cells and its response to insults^48^. To explore the difference in molecular level and to visualise the ET in Cyt *c* redox dynamics for each process, we used generic agents, ethanol and Triton X-100, to induce apoptosis and necrosis in cells, respectively. Although the critical role of Cyt *c* in activating cellular apoptosis has long been verified^30^, previous studies relied on static end point measurements of Cyt *c* redox status. For more detailed information on Cyt *c* redox dynamics, we utilise the A/B/C tunnel junction for cytosolic quantitative QBET imaging of Cyt *c* redox dynamics. The GNPs modified with barrier linker molecules (A/B) were endocytosed by a living cell, and eventually conjugated with intracellular Cyt *c* to form A/B/C complex for QBET measurement.

**Fig. 4:**
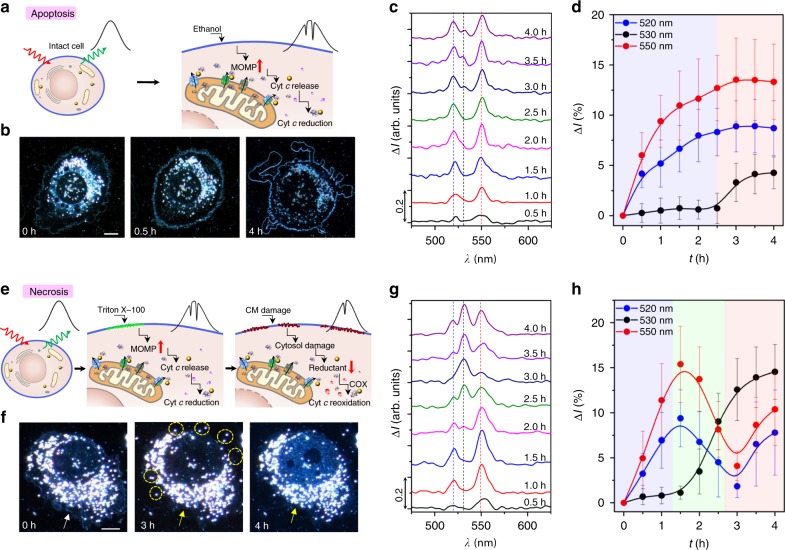
Real-time intracellular QBET imaging of ET during cellular apoptosis and necrosis. **a**–**d**, Non-invasive QBET imaging for cell apoptosis. **a** Schematic of cellular stimulus with ethanol for apoptosis. Major QBET signals for Cyt *c* (red.) are captured during apoptosis. MOMP: mitochondrial outer membrane permeabilisation. **b** Dark-field images of the cell at different time with the stimulus of ethanol, scale bar: 10 μm. *t* = 0 h, the beginning of apoptosis measurement. *t* *=* 0.5 h, cell shrinkage was observed. *t* *=* 4 h, nucleus was ruptured and apoptosis bodies was formed. **c** Scattering spectra difference showing QBET in A/B/C tunnel junction in the process of cell apoptosis, data obtained from region VIII in Supplementary Fig. 9. The QBET spectra differences were obtained by subtracting the spectra at different measuring times from the spectra at the beginning of the measurement (*t* = 0 h). **d** Quantised dips at 520, 530 and 550 nm in the process of cell apoptosis. **e**–**h** QBET imaging with triton X-100 stimulus for cell necrosis. **e** Schematic of cellular stimulus with triton X-100 for necrosis. QBET is captured from Cyt *c* (red.) to Cyt *c* (Ox.) during necrosis. CM: cell membrane, COX: Cyt *c* oxidase. **f** Dark-field images of the cell with the stimulus of triton X-100. *t* = 0 h, white arrow indicates the intact cell membrane; *t* = 3 h, yellow arrow indicates partial lysis of cell membrane, yellow circles indicate organelles are released out to extracellular environment; *t* = 4 h, yellow arrow indicates the damage and lysis of the whole membrane. Scale bar: 10 μm. **g** Scattering spectra difference showing QBET in the process of cell necrosis, data obtained from GNP in region I in Supplementary Fig. 14. The dip changes show the change from Cyt *c* (Red.) to Cyt *c* (Ox.), and finally both Cyt *c* (Red.) and Cyt *c* (Ox.). **h**, Quantised dips at 520, 530, and 550 nm in the process of cell necrosis with cyclic behaviour. The curves in **d**, **h** are cubic B-spline connection of the experimental data (Supplementary Note 6). The cartoons for Cyt *c* molecules in **a**, **e** were created from the RSCB protein data bank^54^. Error bars represent standard deviation

In intact cells, Cyt *c* resides in the inner mitochondrial membrane, and cytosolic Cyt *c* level is very low, thus we are unable to detect any QBET induced dips at the scattering spectra of the GNP (Fig. 4a). Upon apoptotic stimulus via ethanol treatment, mitochondrial outer membrane permeabilisation is increased via outer mitochondrial membrane pore or channel formation, which promotes the release of Cyt *c* from mitochondria. The released Cyt *c* is quickly reduced by cytosolic reductants and enzymes. Accordingly, the QBET in A/B/C (Red.) tunnel junction with dips at 520 and 550 nm were captured. Distinct morphologic changes were observed during apoptosis process, including cell shrinkage, cell membrane blebbing to form apoptotic bodies, and nucleus rupture (Fig. 4b and Supplementary Fig. 8), which are the characteristic features of apoptosis^49^. Significant changes in the QBET signals were also captured in the cytosol after the ethanol exposure (Fig. 4c). There is a heterogeneity in the signals detected from different GNPs at different intracellular locations (Supplementary Figs. 9–11) due to different intracellular environments, the concentration of released Cyt *c*, redox states of Cyt *c*, as well as the amount of Cyt *c* conjugated to GNPs. Fig. 4d shows the dip changes in the whole process of apoptosis (based on more than 80 gold nanoparticles in more than 10 cell repeats). Dips at 520 and 550 nm were captured after 0.5 h of the ethanol exposure, indicating the detection of Cyt *c* (Red.) in the cytosol. After the exposure of 3.0 h, a small dip at 530 nm was also captured. The dip depths became stable after about 3.5 h of the exposure with an average intensity difference (Δ*I*: dip changes compared to values at 0 h) of about 8.9%, 4.2% and 13.5% at 520, 530, and 550 nm, respectively. These results show that in addition to Cyt *c* (red.), Cyt *c* (Ox.) also exists in cytosol at the late stage of apoptosis. At this late stage, the nucleus was ruptured (Supplementary Fig. 8), and the cytosolic reductants might be exhausted. Many studies show that Cyt *c* (Ox.) is more active for caspase-9 activation and apoptosome assembly for apoptosis activation^43^, while another study showed that redox state of Cyt *c* is not a cause but a reporter of cell state during apoptosis^50^. Nonetheless, our real-time QBET imaging reveals that the time at which the redox state of Cyt *c* is measured is critical. At the time point when the apoptotic stimulus applied, the cell still stays intact and healthy, thus the released Cyt *c* is quickly reduced in the cytosol by cytosolic reductants and enzymes. However, cells become apoptotic after 3 h of apoptotic stimulus and Cyt *c* (Ox.) released from mitochondria also exists in the cytosol.

Different plasmonic nanoparticles (silver nanospheres and gold nanorods) were also tested for capturing QBET signals during apoptotic stimulus. However, only the 50 nm GNPs had the necessary overlap between its plasmonic resonant peak and the absorption peak of the conjugated molecule (Supplementary Fig. 12).

Although both apoptosis and necrosis eventually cause cell death, the molecular dynamics of Cyt *c* during these two processes are presumably different due to the difference in inherent biological mechanisms. We stimulated cells with Triton X-100 (0.17 mM) to induce necrosis and utilised QBET junction spectroscopy to image Cyt *c* redox dynamics in the process of necrosis (Fig. 4e). In contrast to apoptosis where the cell membrane was kept intact, the cell membrane was gradually damaged and cellular organelles were released outside the cell during necrosis (Fig. 4f and Supplementary Fig. 13). Significant changes in real-time QBET signals at different locations inside the cell were also captured with Cyt *c* redox changes (Fig. 4g and Supplementary Figs. 14–16). Heterogeneity due to different cellular environments, Cyt *c* concentrations and total amount of conjugated Cyt *c* contribute to different dip depths detected in QBET signals. Dip depth measurements are also affected by changes in redox states of Cyt *c*, which showed clear differences in the dip depths at different wavelength positions during necrosis (530 nm for oxidised Cyt *c*, 520 and 550 nm for reduced Cyt *c*). Figure 4h shows dip changes in the necrosis process based on more than 10 cell repeats, with three different time-dependent distribution of dips (Cyt *c* redox) in a cyclic behaviour. Due to the different dynamics of enzymatic oxidation and reduction inside the cell, this cyclic behaviour of Cyt *c* redox is different from the situation in apoptosis. Dips at 520 and 550 nm were captured after 0.5 h of the exposure, indicating the detection of Cyt *c* (Red.). This initial stage of necrosis is similar to what has been observed for the first stage of apoptosis where cell stays intact and healthy and the released Cyt *c* is quickly reduced by cytosolic reductants. The dips were gradually changed to 530 nm after 2.0 h of the exposure, indicating the reoxidation of Cyt *c*. This is the second stage of necrosis in which increasing in the mitochondrial outer membrane permeabilisation exposed Cyt *c* to COX and reoxidation occurred. At ~3.0 h of the exposure, the dips at 520 and 550 nm reached a minimum, indicating maximum reoxidation. At this stage, the cell membrane was damaged and organelles were released into the extracellular environment (Fig. 3h, 4f). The internal reductive environment was destroyed and the release of COX from mitochondria exhausted the capacity of cytosolic reductants. These factors result in an increased oxidised cellular environment, allowing maximum oxidation of Cyt *c*. After 3.5 h of the exposure, the dips at 520 and 550 nm appeared again. This is due to cell rupture via continuous treatment of Triton X-100 and the mitochondrial outer membrane permeabilisation induced the release of both Cyt *c* (Red.) and Cyt *c* (Ox.) from mitochondria.

The cellular environment provides reductive enzymes to reduce Cyt *c* in the cytosol. Without this reductive environment, we hypothesise that the released Cyt *c* from mitochondria should be found in both oxidised and reduced forms. To show that the detected signal at 520, 530 and 550 nm in the final stage of both apoptosis and necrosis was a result of the release of both Cyt *c* (Ox.) and Cyt *c* (Red.) from mitochondria, we performed similar QBET imaging at the surface of mitochondria using mitochondria extracts. Similarly, the dips at 520, 530 and 550 nm were captured at mitochondria surface (Supplementary Fig. 17) due to the release of both Cyt *c* (Ox.) and Cyt *c* (Red.). Our data indicates that both oxidised and reduced forms of Cyt *c* reside in the mitochondria and the detection of any redox form greatly depends on the internal cellular environment.

## Discussion

Although we utilised PRET technique for the optical detection of Cyt *c* in live cells before^51,52^, we were not able to discern the ET dynamics of Cyt *c* in live cells. In this study, we demonstrated not only the experimental evidence of quantum electron tunneling in the junction, but also pioneering biological experiments using the QBET junction as a biological reporter to distinguish the ET dynamics in the processes of cellular apoptosis and necrosis. The results demonstrate that QBET junction spectroscopy is a powerful method to detect specifically the oxidative state from the reductive state of Cyt *c*, dynamics of ET, and its ability to report the relative concentration of Cyt *c* through the depth of the quantised resonant dips. Redox oscillation determines cellular functions and plays critical role in cancer as well, and there are many hypotheses of biological oscillations in metabolic redox reactions^53^. However, there is no real-time method for ET imaging during redox oscillations non-invasively. The beauty of QBET junction spectroscopy is its ability to distinguish different states (i.e. oxidation and reduction), and ET dynamics during redox oscillations due to the different enzymatic activities in cell death process through the non-invasive molecular imaging of Cyt *c*. It is noted that plasmonic resonance peak can be shifted due to particle aggregation, but the quantised dips of QBET signals are still detectable if the scattering spectra show an overlap with Cyt *c* absorption peaks (Supplementary Fig. 17b and Supplementary Note 5). It is also noted that only a fraction of GNPs endocytosed by the living cell form functional A/B/C tunnel junctions for QBET detection. No dip signatures were detected for unconjugated GNPs while the detection of different dip signatures (Supplementary Fig. 18) indicates the conjugation of other molecules to the GNPs. However, we are unable to identify the biomolecules corresponding to the different dips in the present study. Therefore, we limit the scope of this study to detecting Cyt *c* with clear quantised dips at different redox states. However, the QBET technique shows potential for the discovery and detection of various biomolecules in living cells. Based on the observation of more than 20 cells and 200 GNPs, we found that about 48% of GNPs form functional A/B/C tunnel junctions for QBET detection of Cyt *c* in living cells.

We obtained the first quantum biological electron tunnelling in live cells and living enzymes. The non-invasive and real-time optical imaging of ET dynamics of Cyt *c* in living cell during the cell death process was accomplished by the QBET junction spectroscopy. The QBET with different tunnelling barrier widths was demonstrated in A/B/C tunnel junction. We captured the real-time QBET signal during Cyt *c* reduction process and the different redox dynamics during cell death process. We experimentally verified the theoretical conjecture of biological ET using QBET junction spectroscopy in particular in live cells. We are now able to optically capture the quantum electron tunneling in living enzymes in a wireless and noninvasive manner. QBET junction spectroscopy pushed the boundary of live cell imaging to overcome the limitations of traditional ET imaging methods, which are invasive and unfeasible for living cell. Although we only show the capture of ET in live cells using Cyt *c*, this technique provides many new possibilities for the capture of biological ET in other enzymes as well as other biomolecules in living biological systems. This direct visualisation of quantum electron tunneling demonstrated in this study has many potentials for the future studies of quantum electron transfer in biological systems, and provide new insight into the quantum mechanisms in governing cellular processes as well as life and death. The ability of QBET junction spectroscopy to distinguish between apoptosis and necrosis is also fundamental as apoptotic death is controlled while necrotic death can cause inflammation and septic shock to the body. Visualisation of real-time ET processes and molecular profiles with temporal and spatial occurrence in live cells with QBET junction spectroscopy will also lead to new avenues for precision molecular diagnostics and therapeutics. It could also provide new insight into the fundamental driving force of biological oscillations due to the different reduction and oxidation of enzyme in living systems, which are the most critical information for the energy harvesting and healthy life.

## Methods

### Experimental setup

The experimental setup is based on an inverted dark-field microscope and a monochrometer. The dark-field microscope consists of a Nikon inverted microscope (Eclipse Ti2, Nikon, Japan) equipped with a dark-field condenser (Oil type, 1.20 < NA < 1.43, TI-DF), a white light source (Halogen Lamp), and three objective lens (CFI TU Plan FLUOR BD 20 × , NA/WD: 0.45/4.5 mm; CFI TU Plan FLUOR BD 50 × , NA/WD: 0.80/1.00 mm; and CFI S Fluor 100X oil with Iris diaphragm, NA: 0.5–1.30, WD: 0.20). The monochrometer is a 303 mm focal-length monochrometer (Andor Shamrock 303i, Andor Technology Ltd, Belfast, Northern Ireland) equipped with a 1600 × 200-active pixel cooled spectroscopic EMCCD camera (Andor Newton 970, Andor Technology Ltd, Belfast, Northern Ireland). An aperture with a changeable width of about 0.5~3 μm was placed in front of the entrance side input slit of the monochrometer to insure only a single GNP appeared in the *x* direction, while in the *y* direction, 200 pixels of individual spectra for different number of particles can be obtained at one time. The scattering spectra were acquired using Andor SOLIS (Andor Technology Ltd, Belfast, Northern Ireland). The optical power of the light source irradiated on the sample is changeable from 0 to 200 µW cm^−2^. The integration time for image and spectral acquisition is changeable from 1 to 500 ms and from 0.1 to 1 s, respectively, depending on the applied light intensity.

### Sample preparation for quantum tunnelling behaviour study

Glass slides were cleaned in a piranha solution (20 min). A cleaned glass slide was then modified with 3-mercaptopropyl-trimethoxy-silane (MPTMS, Sigma-Aldrich) to form a surface with thiol groups, which was realised by incubation in 1 mM MPTMS isopropyl alcohol (IPA) for 24 h. The glass slide was then rinsed with acetone and IPA, and dried with clean nitrogen gas. Fifty-nanomtre spherical GNPs (Sigma-Aldrich) were immobilised on the MPTMS-modified glass slide by the thiol groups (24 h). The GNP surface was then modified with linker molecules of carboxyl-terminated alkanethiol HS(CH_2_)_*n*_COOH (*n* = 2, 3, 5, 7 and 10) and aromatic linker molecules HS(C_6_H_4_)_*n*_COOH (*n* = 1 and 2) by incubation in 1 mM linker molecules IPA for 24 h. One side of the linker molecules were conjugated to GNP surface by thiol groups. The linker molecule-modified GNPs were then incubated with Cyt *c* (from bovine heart, Sigma-Aldrich) in phosphate buffered saline (PBS, Lonza) (50 μM) for 0.5 h, which ensure that Cyt *c* conjugated with GNPs via the linker molecules reached a saturated state^51^. The commercially available Cyt *c* was in the oxidised form. The Cyt *c* (Red.) was obtained by adding excess sodium dithionite (Na_2_S_2_O_4_) into Cyt *c* (Ox.) solution in deoxygenated PBS buffer. Both Cyt *c* (Red.) and Cyt *c* (Ox.) (50 µM) were then used for QBET imaging.

### QBET imaging in Cyt *c* reduction process

To realise the real-time QBET imaging in A/B/C tunnel junction during Cyt *c* reduction, we used excess sodium dithionite (Na_2_S_2_O_4_, 10 mM) as the reductant, and carried out the experiments with Cyt *c* solution (in phosphate buffered saline, PBS, buffer) in vitro. The GNP modified with linker (barrier) molecule (A/B) conjugates with Cyt *c* to form A/B/C complex. In general, a drop of 50 μM Cyt *c* (Ox.) (5 μL, in PBS buffer) was first added to the MPA-GNP modified glass slide, and then a drop of 10 mM Na_2_S_2_O_4_ in deoxygenated PBS with the same volume (5 μL) was added next to the Cyt *c* (Ox.) drop. The sample was covered with a clean cover slide and undergo QBET capture via dark-field microscopy. QBET in A/B/C (Ox.) was first captured due to the conjugation of Cyt *c* (Ox.) to GNP. Cyt *c* reduction started after about 13 min, due to the approach of dithionite anion (S_2_O_4_^2−^) to GNP conjugated with Cyt *c* (Ox.) by Brownian motion.

For Cyt *c* reduction in bulk solution, Cyt *c* (Ox.) (50 μM) was mixed with Na_2_S_2_O_4_ in deoxygenated PBS (10 mM). Real-time absorption spectrum was immediately captured using a UV-Visible spectrophotometer (Tecan Spark Spectrophotometer, Austria) after mixing of bulk solution to observe Cyt *c* reduction.

### Intracellular QBET imaging

MPA-modified GNPs were first prepared for cellular uptake. In general, GNPs (3.5 × 10^10^ mL^−1^) were mixed with MPA (0.1 mM) in PBS buffer for 12 h. Excess MPA and anions were removed from MPA-modified GNPs by centrifugation for 15 min at 4500 rpm twice, and resuspended in PBS buffer. 1600 Hela cells were seeded per cell culture insert (Ibidi) on poly lysine coated slides (Thermo Scientific), grown overnight in Dulbecco’s Modified Eagle Medium (DMEM) supplemented with 4500 mg L^−1^ glucose, 10% fetal bovine serum (FBS) and 1% Penicillin/streptomycin (Pen/strep) at 37 °C, 5% CO_2_ water-jacketed incubator. To avoid the non-specific binding of proteins in FBS to GNP surface, cells were washed with DMEM without FBS and Pen/strep before incubation with MPA-modified GNPs. Cells were then cultured overnight with DMEM without FBS and Pen/strep, mixed with MPA-modified GNPs (~10^9^ particles mL^−1^). Cells were washed with PBS twice to remove the excess GNPs at cell surface. After washing, 3.0% ethanol (in DMEM with FBS and Pen/strep) was added for apoptosis stimulus. For necrosis stimulus, 0.17 mM Triton X-100 (Sigma-Aldrich) (in DMEM with FBS and Pen/strep) was added. Intracellular imaging during cellular apoptosis and necrosis was carried out under dark-field microscopy. Methods described above was used for QBET imaging using silver nanoparticles (AgNPs,10 nm, Sigma-Aldrich) and gold nanorods (GNRs, 25 × 60 nm, Sigma-Aldrich). To avoid the photothermal effect due to the long exposure time from the light source to the cell, white light was illuminated to the sample only when the measurements were performed.

### QBET imaging in mitochondria extracts

Mitochondria extracts were prepared using the Mitochondria Extraction Kit, human (Miltenyi Biotec, Bergisch Gladbach, Germany) according to manufacturer’s instructions. Hela cells were grown to 75% confluency, harvested and lysed in ice-cold Lysis Buffer at 10^7^ cells ml^−1^ passing through 27 G needle (Becton Dickinson). Mitochondria were magnetically labelled with 50 µl of anti-TOM22 MicroBeads in separation buffer for 1 h at 4 °C. The suspension was then loaded onto a LS column (Miltenyi Biotec), which had been placed in a magnetic cell separation (MACS) Separator. After washing the column with separation buffer, it was removed from the MACS Separator and the magnetically labeled mitochondria were eluted with 1.5 mL separation buffer. The mitochondria eluate was centrifuged at 12,000 g for 3 min at 4 °C and resuspended in PBS. The mitochondria extracts were then mixed with MPA-modified GNPs (~10^9^ particles mL^−1^) for 2 h at 4 °C, and used for QBET imaging.

### Reporting summary

Further information on research design is available in the Nature Research Reporting Summary linked to this article.

## Supplementary information

Supplementary Information

Description of Additional Supplementary Files

Supplementary Movie 1

Reporting Summary

## Data Availability

The data that support the findings of this study are available from the corresponding author upon reasonable request.
