# Supplementary material for: Quantum biological tunnel junction for electron transfer imaging in live cells
Source: Nat Commun. 2019 Jul 19;10:3245. doi: 10.1038/s41467-019-11212-x (PMC6642182; doi:10.1038/s41467-019-11212-x)
Supplement: Supplementary file 1 — Supplementary Information [file 41467_2019_11212_MOESM1_ESM.pdf]

Supplementary Information for

Quantum biological tunnel junction for electron transfer imaging in live cells

Xin *et al.*

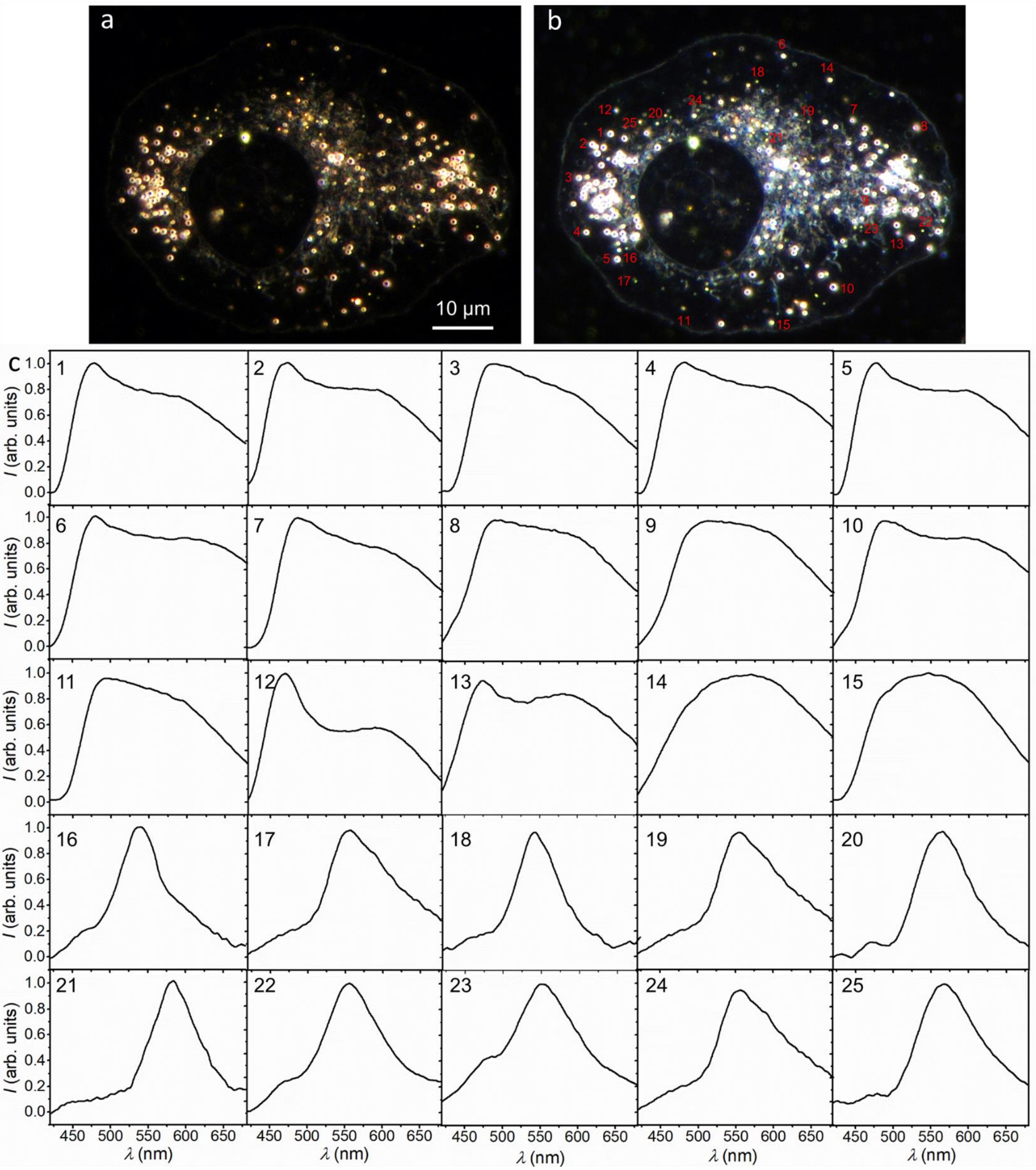

Supplementary Figure 1 | Characterization of organelles and GNPs inside intact Hela cell. (a,b) Dark-field image of a Hela cell with GNPs inside. (a) Image with an exposure time of 100 ms (light source power: 45 μW cm^{-2}), clearly showing the organelles. (b) Image with an exposure time of 200 ms (light source power: 45 μW cm^{-2}). The numbers indicate different organelles and particles for spectrum taken in (c). (c)

Scattering spectra of organelles and GNPs as marked in (b). From 1 to 15, the spectra are for organelles and are broad. From 16 to 25, the spectra are for GNPs with plasmonic peaks. The different position of the peaks indicate some of the GNPs are aggregates inside the cell.

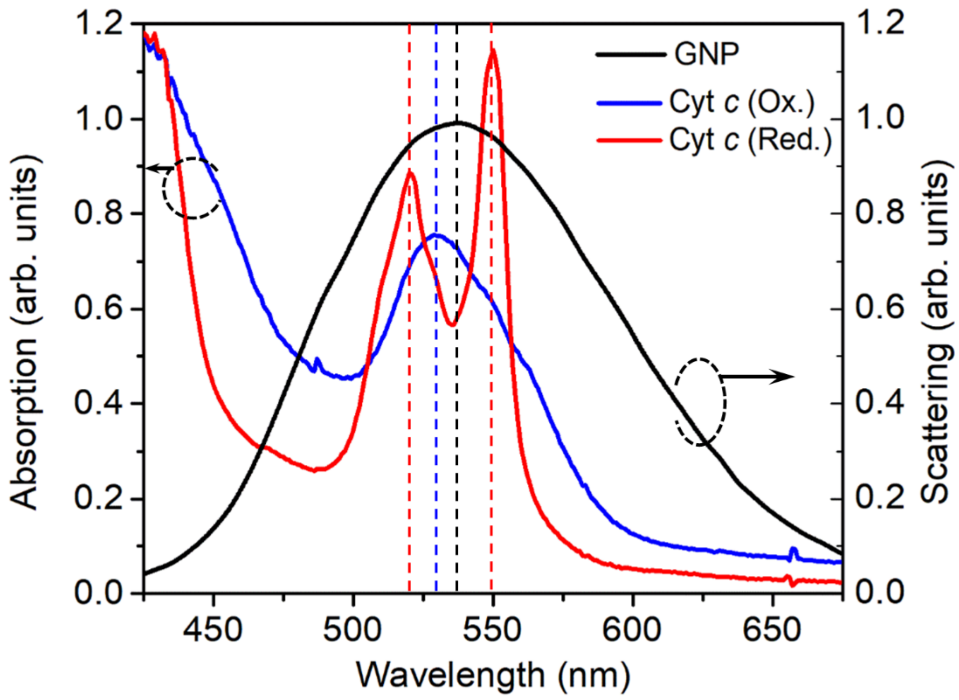

Supplementary Figure 2 | Spectra characterization of Cyt *c* and GNP. The blue and red curves are the absorption spectra for Cyt *c* (Ox.) and Cyt *c* (Red.), respectively, while the black curve is a representative scattering spectrum of a single GNP. The open dashed circle with a leftwards arrow indicates the blue and red curves are linked to the left axis, while the open dashed circle with a rightwards arrow indicates the black curve is linked to the right axis. The peak of Cyt *c* (Ox.) (blue curve) is observed at around 530 nm (dashed blue line), while that for Cyt *c* (Red.) (red curve) are observed at 520 and 550 nm (dashed red line). The plasmonic peak of the GNP is 538 nm (dashed black line).

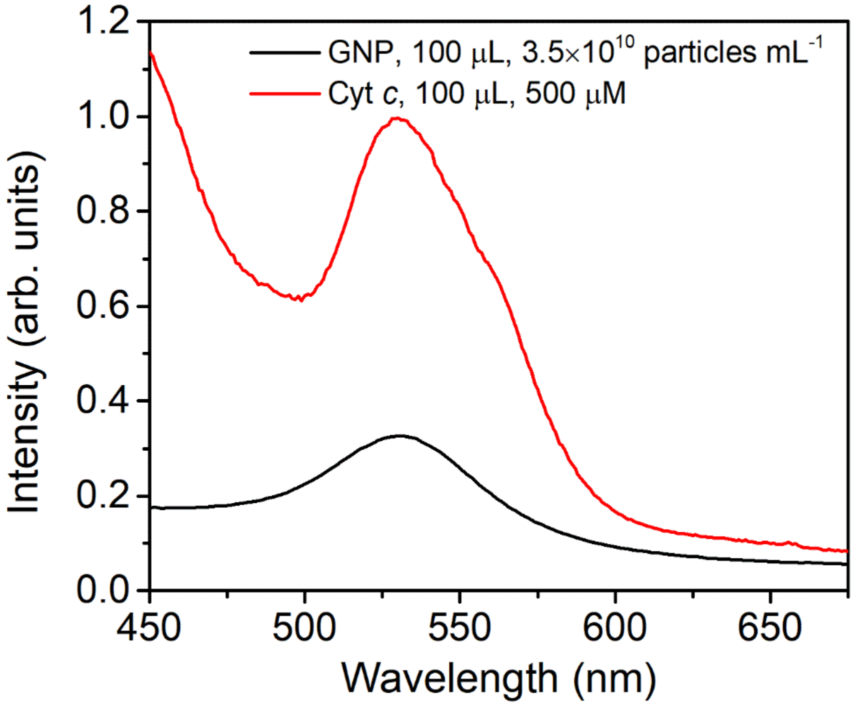

Supplementary Figure 3 | Absorption spectra of GNPs and Cyt *c* (Ox.). To compare the extinction coefficient of a single GNP and a single Cyt *c* molecule, we have measured the absorption spectra of GNPs and oxidized Cyt *c* in PBS buffer. The concentration of GNPs and Cyt *c* for the measurement were 3.5×10^{10} particles mL^{-1} and 500 μM, respectively. The extinction coefficients were measured based on 100 μL of both solution, with about 3.5×10^{9} GNPs and 3.01×10^{16} Cyt *c* molecules. The measured extinction coefficient of a single GNP is about 6 orders of magnitude higher than that for a single Cyt *c* molecule.

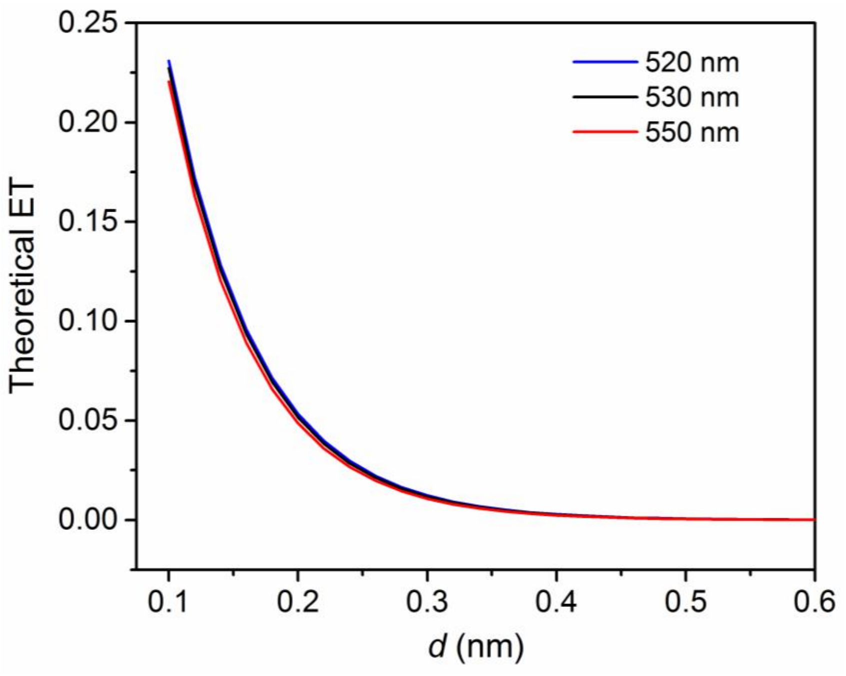

Supplementary Figure 4 | Theoretical calculation of electron tunnelling. Theoretical calculation of a single electron tunnelling behaviour as a function of barrier width for electrons with different energy corresponding to wavelength of 520, 530, and 550 nm. For a single electron, the probability is theoretically given by $P = \exp(-\beta d)$. Based on the calculated smallest LUMO level of −1.07 eV for carboxyl-terminated alkanethiol linker molecules by Hansen *et al.*[1], the smallest potential barrier in the quantum junction is about 4.43 eV when considering the Fermi level of gold of about 5.5 eV. The calculated tunnelling decay rate is β = 1.46, 1.48, and 1.51 $Å^{-1}$ for the electron with respective energy of 2.39, 2.34, and 2.26 eV, corresponding to wavelength at 520, 530, and 550 nm.

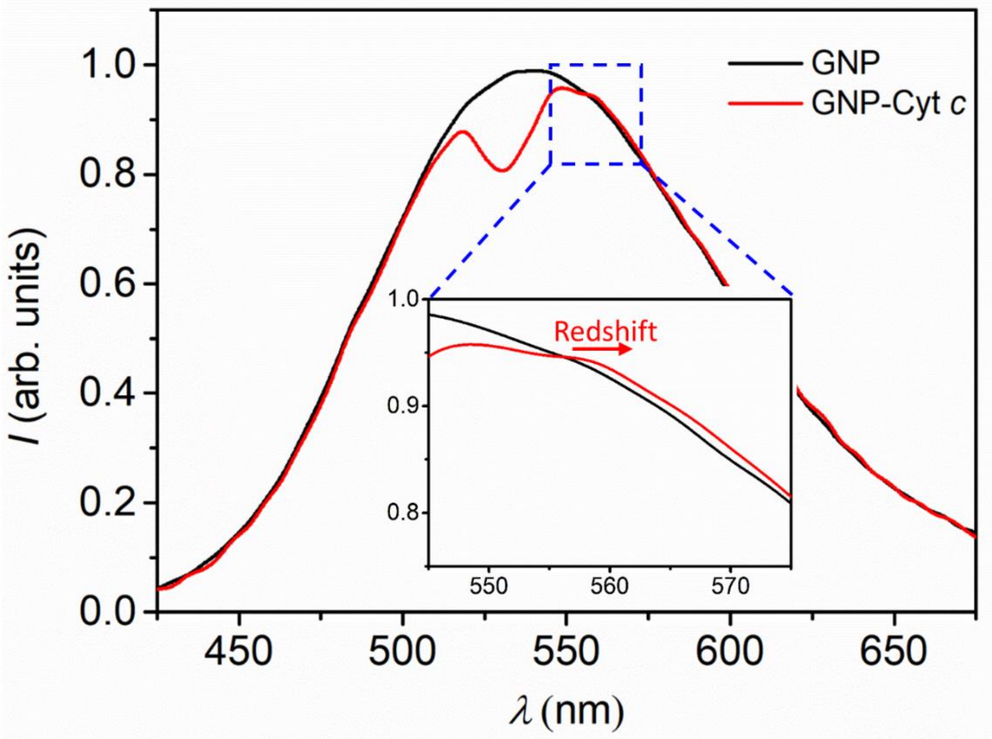

Supplementary Figure 5 | Redshift of the scattering spectrum in the GNP-Cyt *c* conjugation.

Inset shows the zoom-in view of the dashed blue area, showing a small redshift in the spectrum.

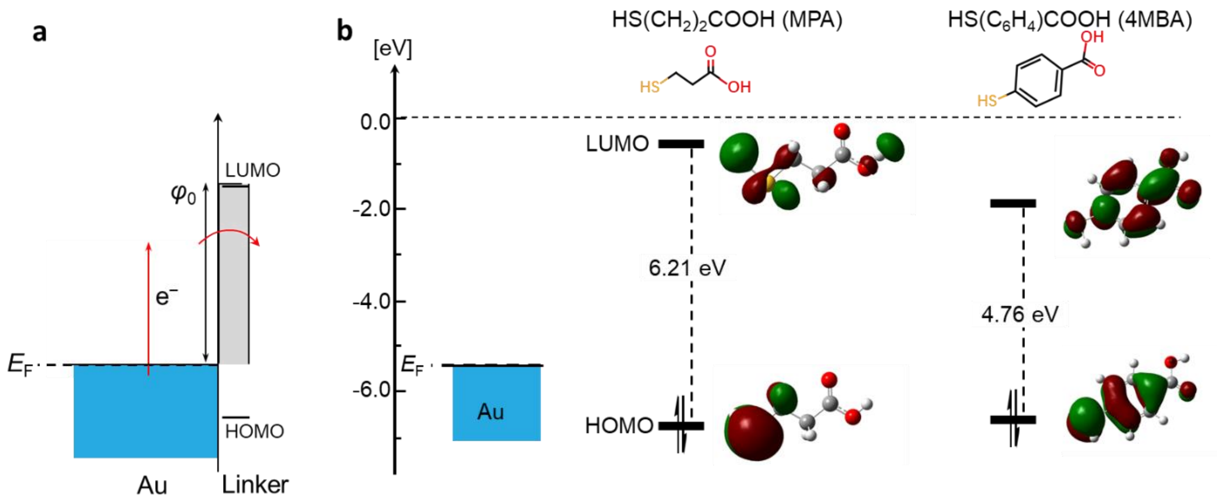

Supplementary Figure 6 | Tunnelling barrier with different linker molecules. (a) Schematic illustration of the tunnelling barrier height through the linker molecule, the barrier height is the offset between the Fermi level (E_F) of gold (Au) and the LUMO of the linker molecule. (b) Calculated energetic and spatial distribution of the HOMOs and LUMOs of two representative linker molecules we used as linker molecules. The aromatic molecules show a lower HOMO-LUMO gap than that of the carboxyl-terminated alkanethiol. The barrier height can be estimated to be 4.93 and 3.65 eV for the tunnel junction formed with MPA and 4MBA, respectively. The calculation is based on the software of Gaussian 09, Revision D.01, using B3LYP/6-311++G**.

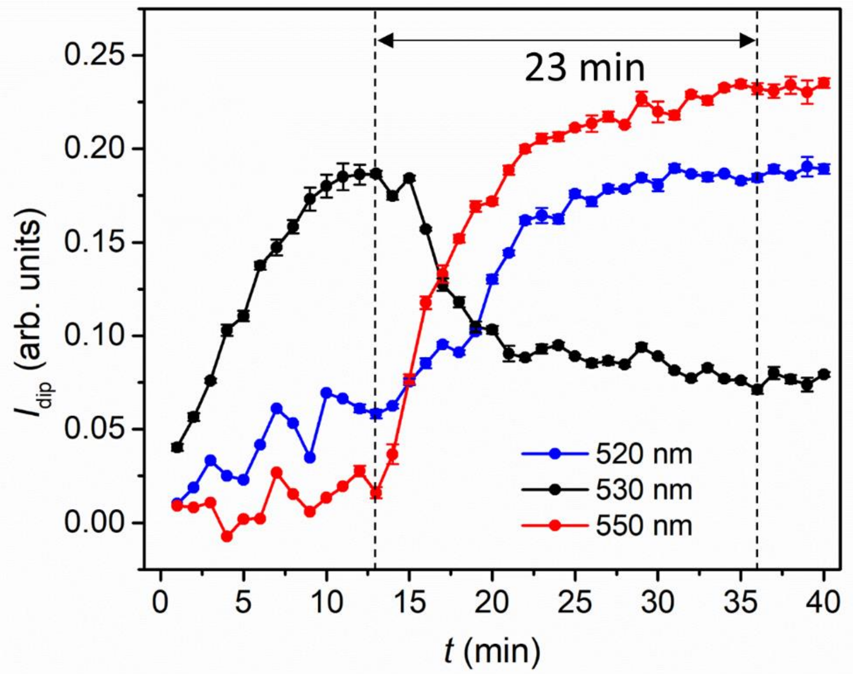

Supplementary Figure 7 | Real-time change of dip depth in conjugation and reduction process. The reduction started at around 13 min, with a sudden increase in the dip depth of 550 nm for Cyt *c* (Red.), and the dips were stable at around 36 min. The whole reduction process took about 23 min.

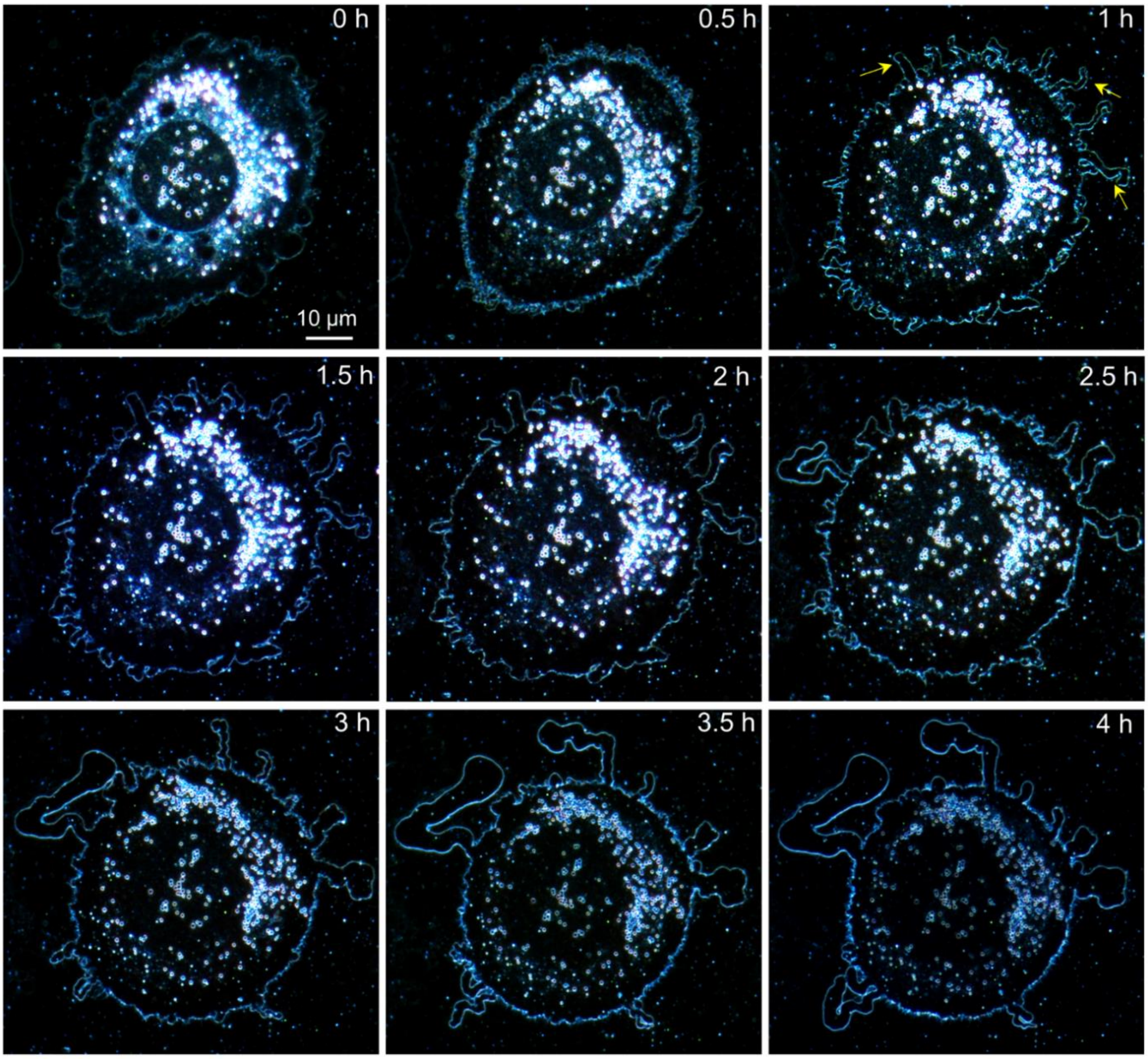

Supplementary Figure 8 | Dark-field images of Hela cell during apoptosis at 0.5 h intervals.

Exposure time of 200 ms, light source power: 45 μW cm^{-2}. Changes in cellular morphology, including both cell membrane and nucleus, was captured during the apoptosis. Within the first 0.5 h, cell shrinkage was observed. At t = 1 h, extensive cell membrane blebbing occurred (yellow arrows indicated). At t = 2 h, nucleus began to break down. From 2 to 4 h, apoptosis bodies were formed by membrane blebbing.

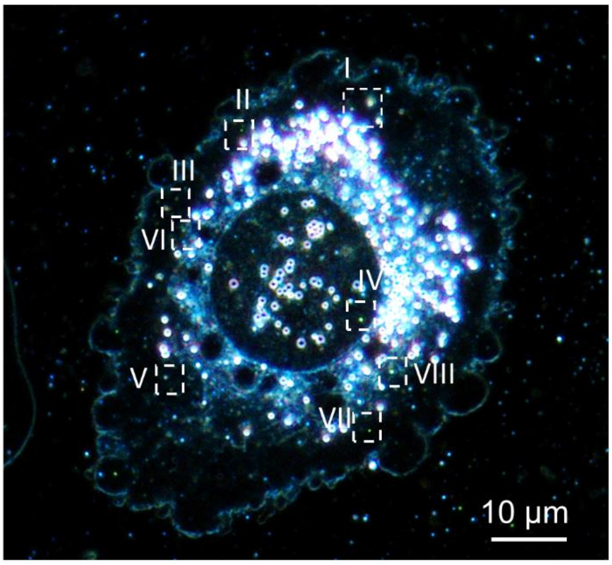

Supplementary Figure 9 | Hela cell with GNPs inside at the beginning of ethanol exposure. Dashed squares indicate 8 different regions of GNPs for QBET imaging during the apoptosis process.

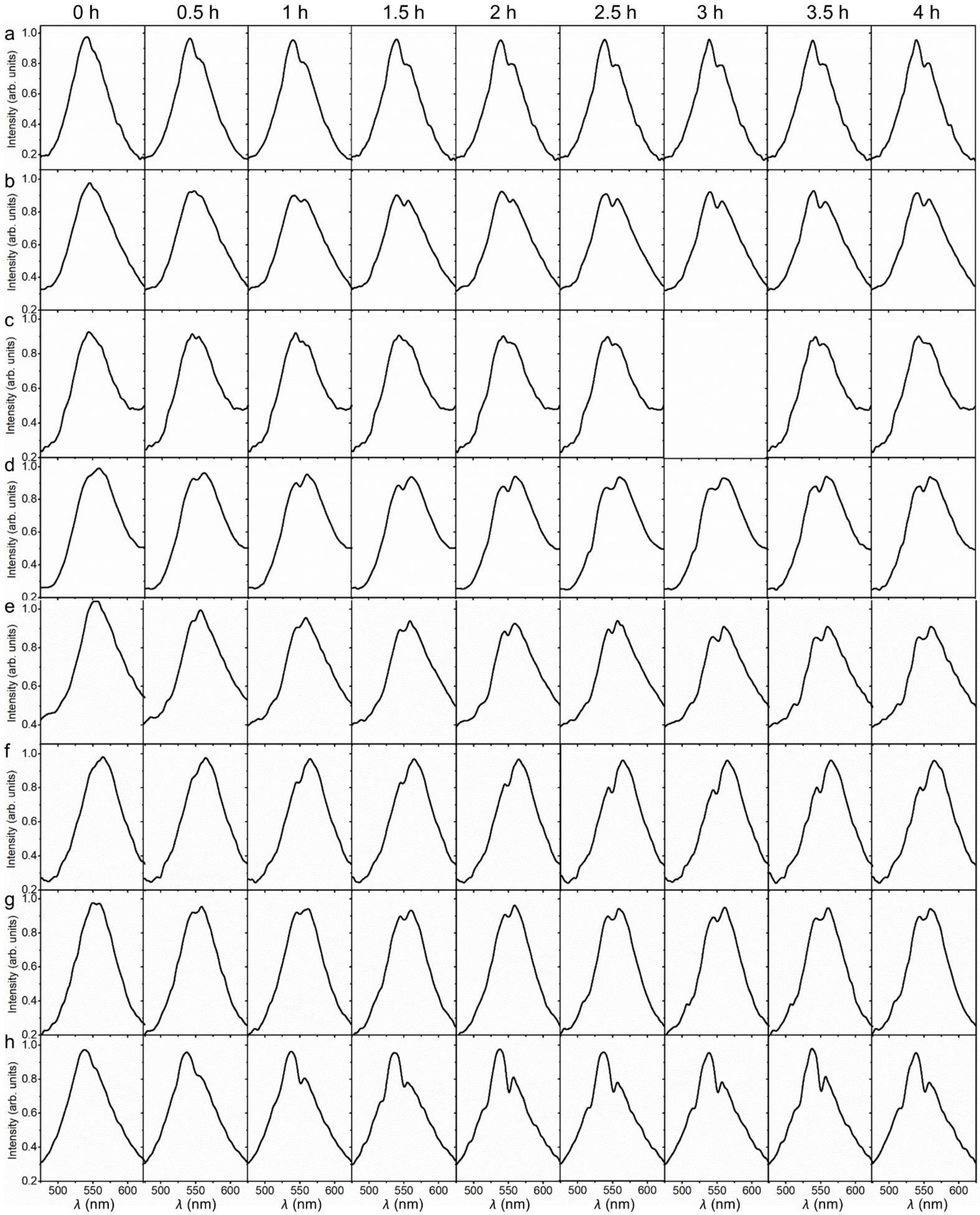

Supplementary Figure 10 | QBET imaging at different regions during apoptosis process. Panels a-h are scattering spectra of GNPs for QBET imaging taken from the regions I-VIII in Supplementary Fig. 9 at 0.5 h intervals, respectively. The blank at $t = 3.0$ for GNP in region III was because the GNP was transiently hidden by the organelle due to Brownian motion, and the spectrum was unable to take.

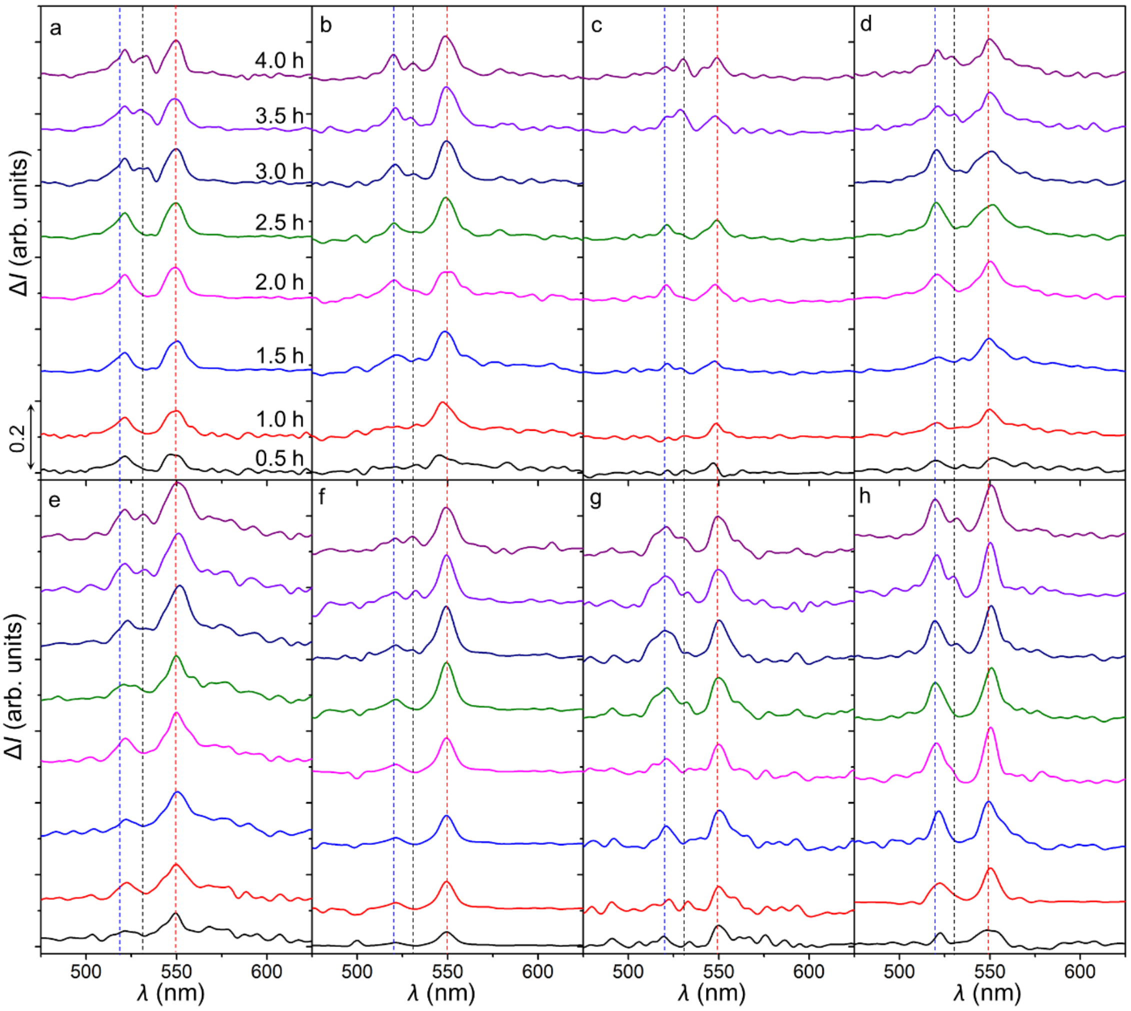

Supplementary Figure 11 | Spectra differences of GNPs at different time of apoptosis. Panels a-h are for the GNPs in the regions I-VIII in Supplementary Fig. 9, respectively. The spectra differences were obtained by subtracting the spectra at different measuring time from the spectra at the beginning of the measurement (t = 0 h). The dashed blue and red lines respectively indicate the peaks (corresponding to spectra dips) at 520 and 550 nm for Cyt *c* (Red.), while the black dashed lines indicate the dips at 530 nm for Cyt *c* (Ox.). The dynamic changes of peaks in the spectra differences show Cyt *c* dynamics during apoptosis. Cyt *c* (Ox.) with the peak at around 530 nm was detected after 3.0 h. The blank at t = 3.0 for GNP in region III was because the GNP was transiently hidden by the organelle due to Brownian motion, and the spectrum was unable to take.

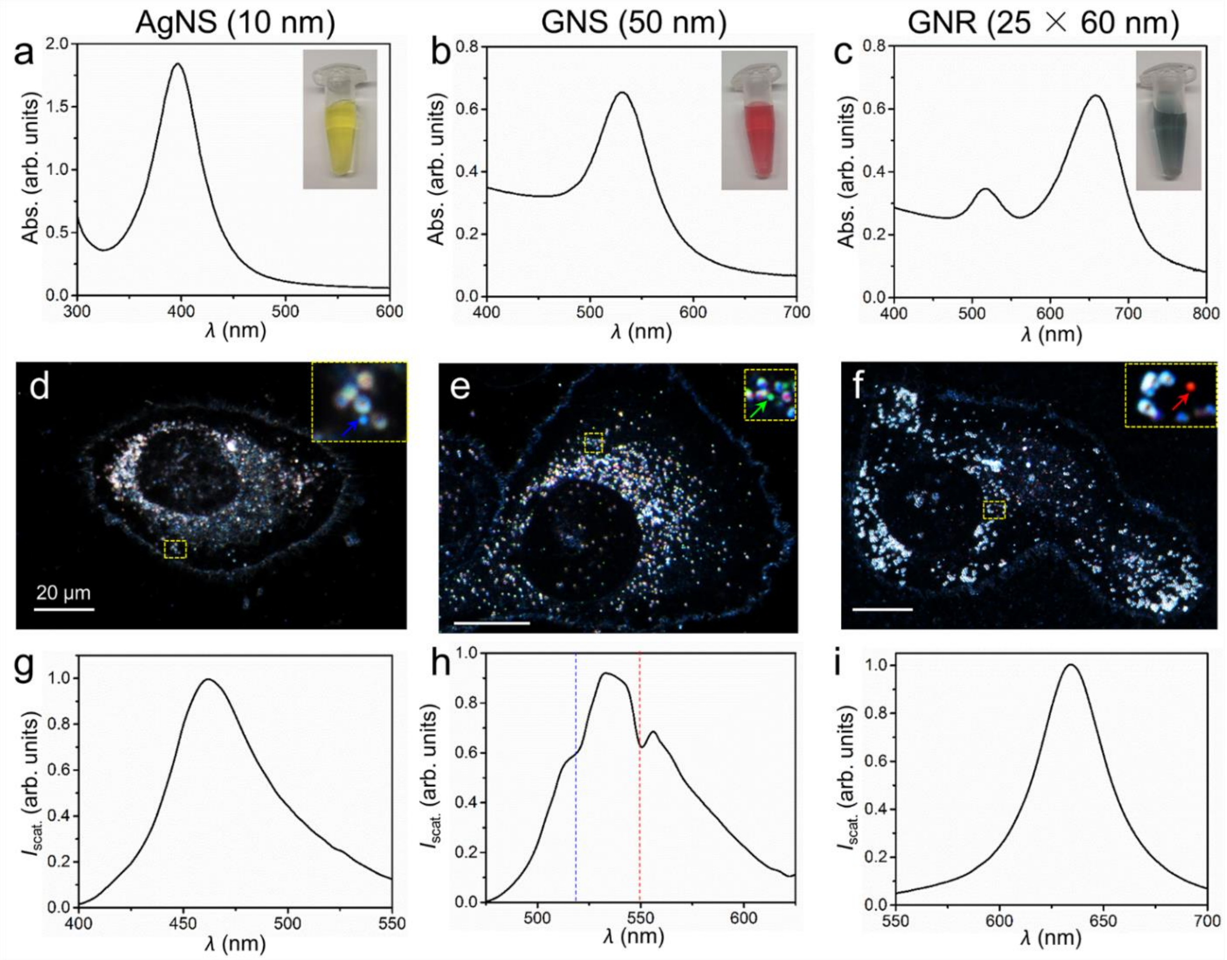

Supplementary Figure 12 | Intracellular QBET imaging using different nanoparticles. (a-c) Absorption of three different nanoparticle solutions of (a) AgNS (10 nm), (b) GNS (50 nm), and (c) GNR (24 × 60 nm), insets show the photographs of nanoparticle solutions. (d-f) Dark-field images of Hela cells with three MPA-modified nanoparticles of (d) AgNS (10 nm), (e) GNS (50 nm), and (f) GNR (24 × 60 nm), insets show the nanoparticles (blue, green, and red arrow indicated) inside the cell. Scale bars: 20 μm. (g-i) Scattering spectra of the corresponding particles in the insets of (d-f) after one hour of apoptosis stimulus. Only GNS (50 nm) was able to capture the QBET signals. That is because the peaks of the other two scattering spectra had no overlap with the absorption of Cyt *c*.

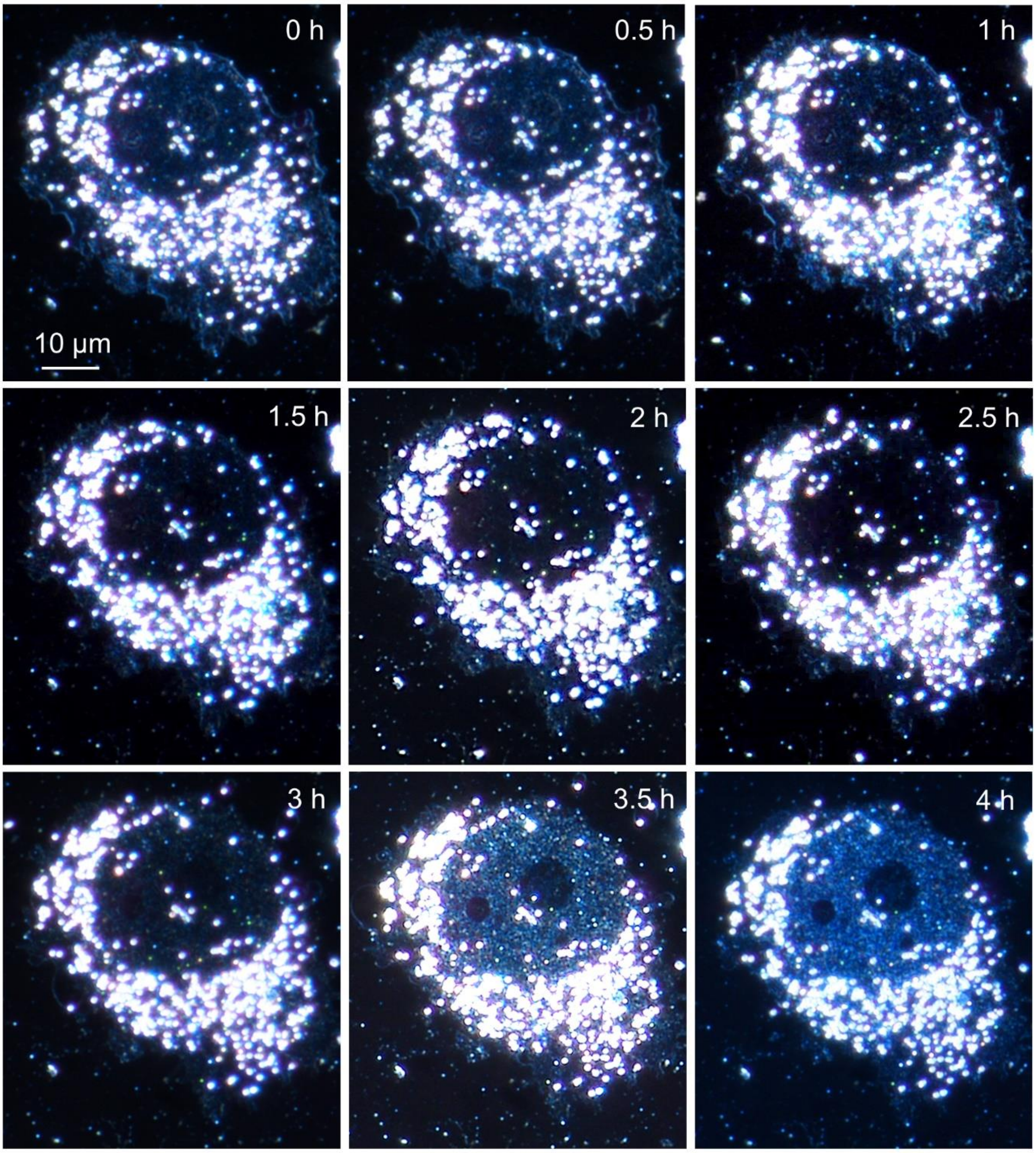

Supplementary Figure 13 | Dark-field images of Hela cell during necrosis at 0.5 h intervals. Exposure time of 200 ms, light source power: 45 μW cm^{-2}. Changes in cellular morphology, including both cell membrane and nucleus, was captured during the necrosis. Membrane damage and organelle release were observed at 2 h, and the cell was totally lysed at 4 h.

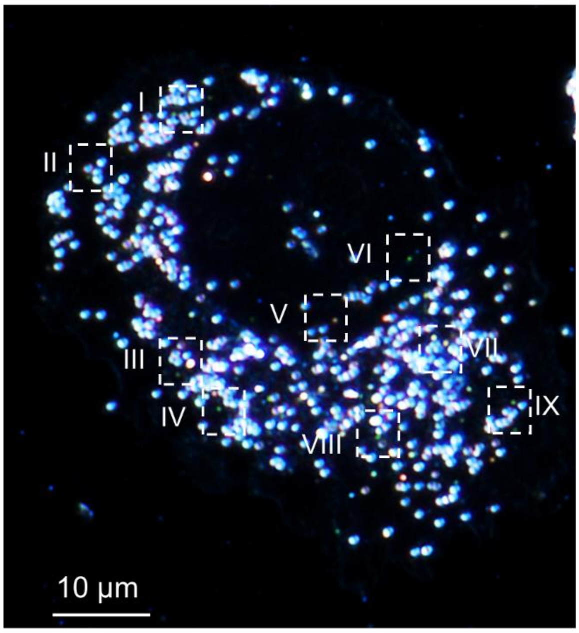

Supplementary Figure 14 | Hela cell with GNPs uptake at the beginning of necrosis. Dashed squares indicate ten different regions for QBET imaging in the necrosis process. To show the GNPs inside the cell, the exposure time was decreased from 200 to 100 ms (light source power: 45 μW cm^{-2}). In that case, the cell membrane was too dark to observe.

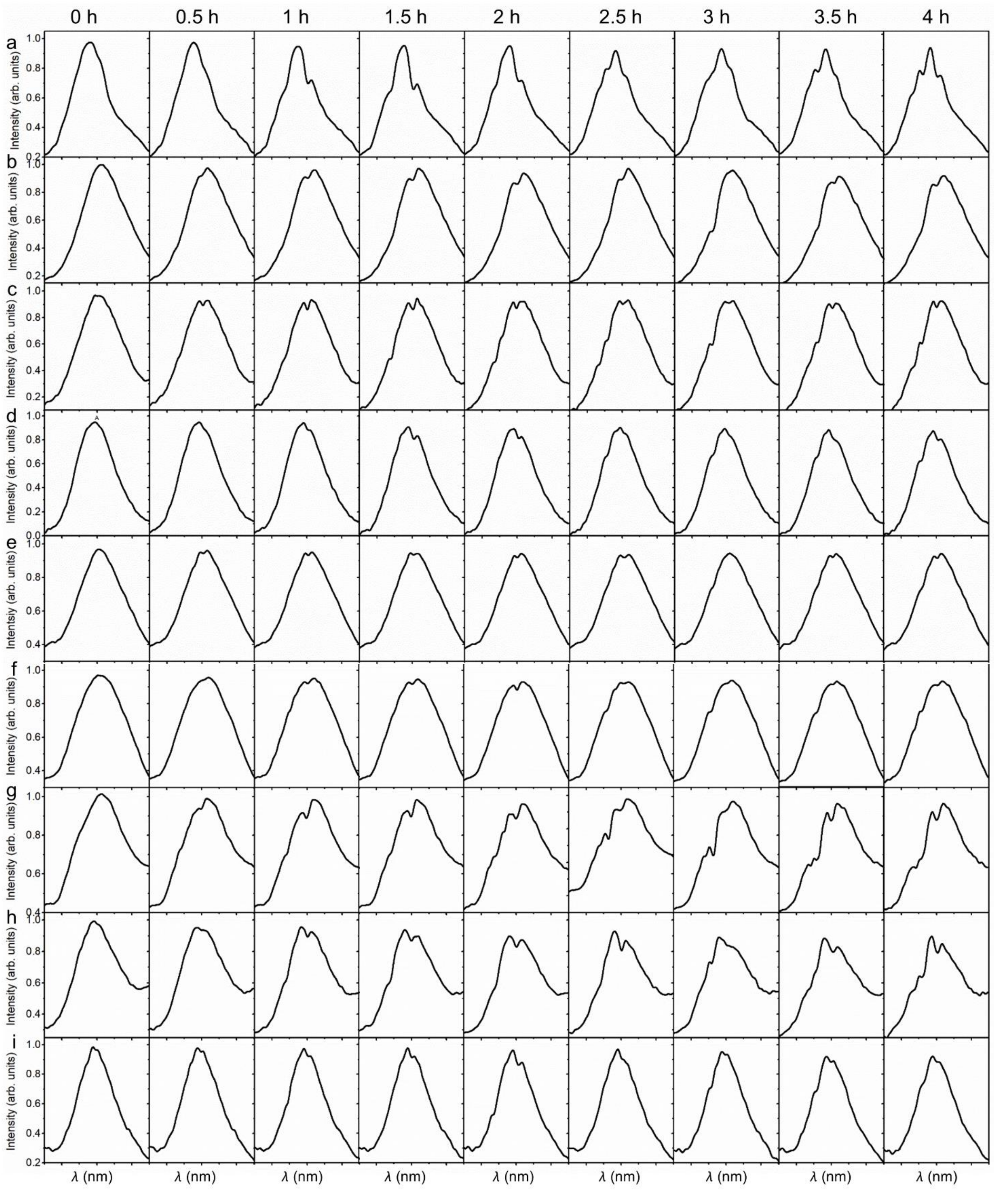

Supplementary Figure 15 | QBET imaging at different regions during necrosis process. Panels a-i are scattering spectra of GNPs for QBET imaging taken from the regions I-IX in Supplementary Fig. 14 at 0.5 h intervals, respectively. The dashed blue and red lines respectively indicate the dips at 520 and 550 nm for Cyt *c* (Red.), while the black dashed lines indicate the dips at 530 nm for Cyt *c* (Ox.).

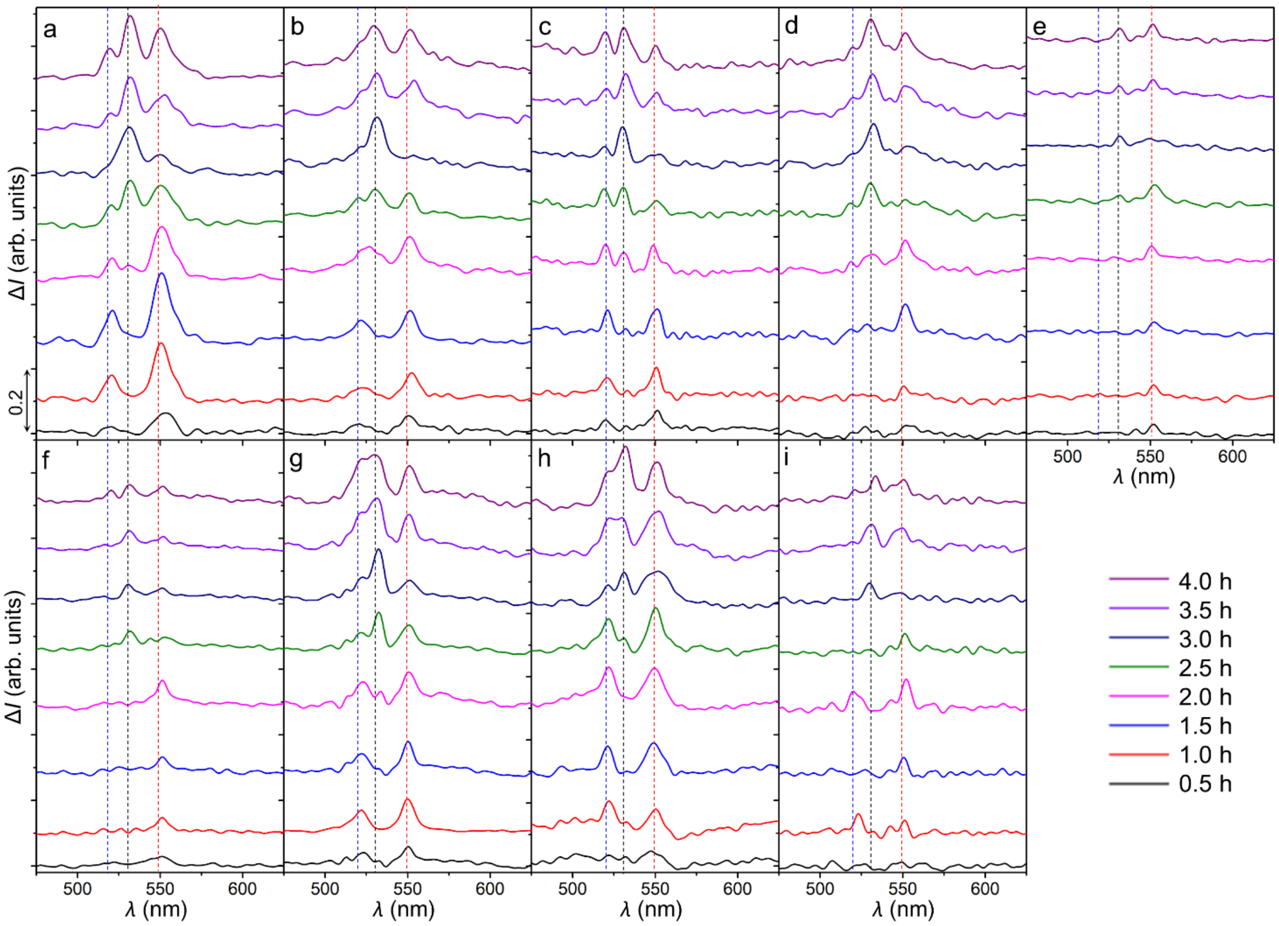

Supplementary Figure 16 | Spectra differences of GNPs at different time of necrosis. Panels a-i are for the GNPs in the regions I-IX in Supplementary Fig. 14, respectively. The dashed blue and red lines respectively indicate the peaks at 520 and 550 nm for Cyt *c* (Red.), while the black dashed lines indicate the peaks at 530 nm for Cyt *c* (Ox.). The dynamic changes of peaks in the spectra difference shows Cyt *c* redox dynamics during necrosis. The change in Cyt *c* redox was in accordance with the change in cell morphology. For example, within 2 h, the detected dips were located at around 520 and 550 nm, these were for Cyt *c* (Red.). In this period, the cell membrane was intact as shown in Supplementary Fig. 13. Dips were changed from 520 and 550 nm to 530 nm after 2 h, indicating Cyt *c* was reoxidised. In this period, the cell membrane was damaged as shown in Supplementary Fig. 13, the cytosol reductants were damaged, which resulted in the reoxidation of Cyt *c* by COX released from mitochondria. All the dips at 520, 530, and 550 were captured at t = 3.5 and 4 h. That is because the cell was totally lysed, and both Cyt *c* (Red.) and Cyt *c* (Ox.) were released from mitochondria.

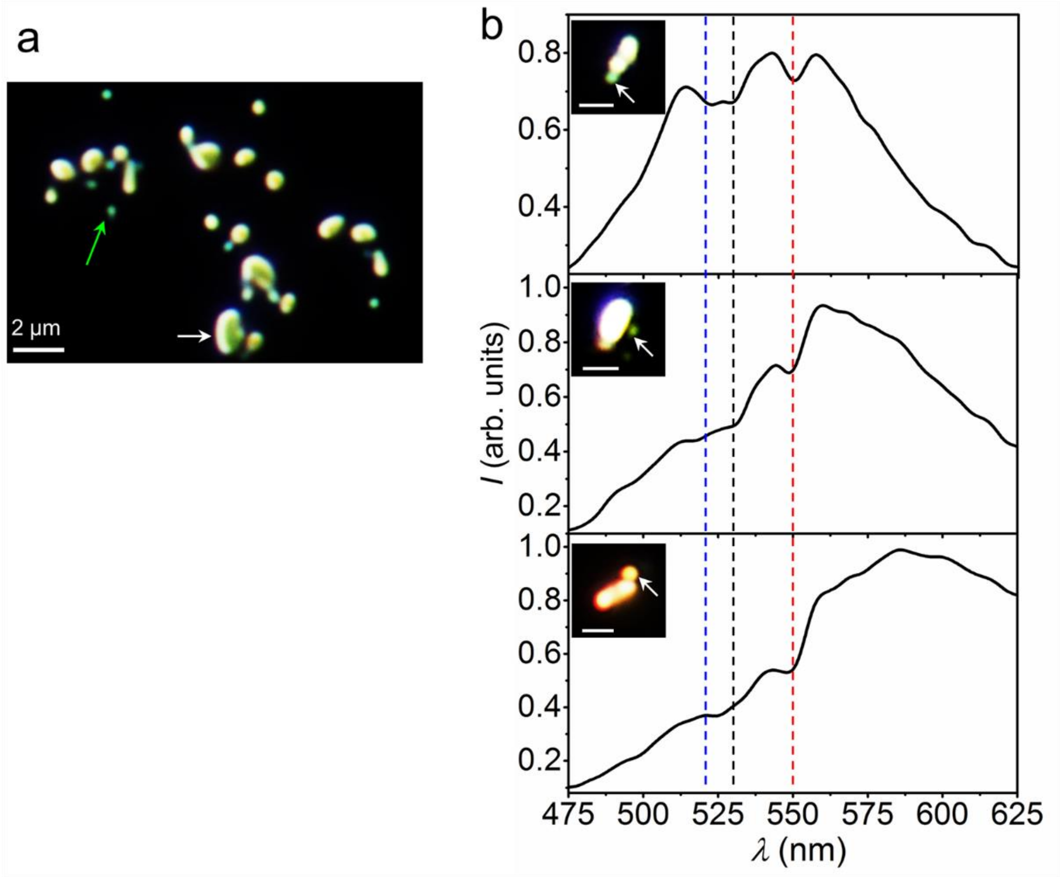

Supplementary Figure 17 | QBET imaging at mitochondria surface. (a) Dark-field image of mitochondria with GNPs, the green and white arrows indicate GNPs and mitochondria, respectively. (b) QBET captured by different size of nanoparticle aggregates which have different plasmonic resonance peaks (λ_{max}) at mitochondria surface. Insets show the dark-field image of the nanoparticle aggregates on the surface of mitochondria, scale bar: 2 μm. The white arrow indicates the nanoparticle aggregate. From I to III, the plasmonic resonance peaks are gradually redshifted from 540 nm, to 560 nm, and to 585 nm. The dashed blue, black, and red lines indicate the dips at 520, 530, and 550 nm of the absorption peak wavelengths of Cyt *c*, respectively. The quantized dip depths gradually attenuate with the redshift of the plasmonic resonance peak. This shows the importance of matching resonances between antennas and biological molecules.

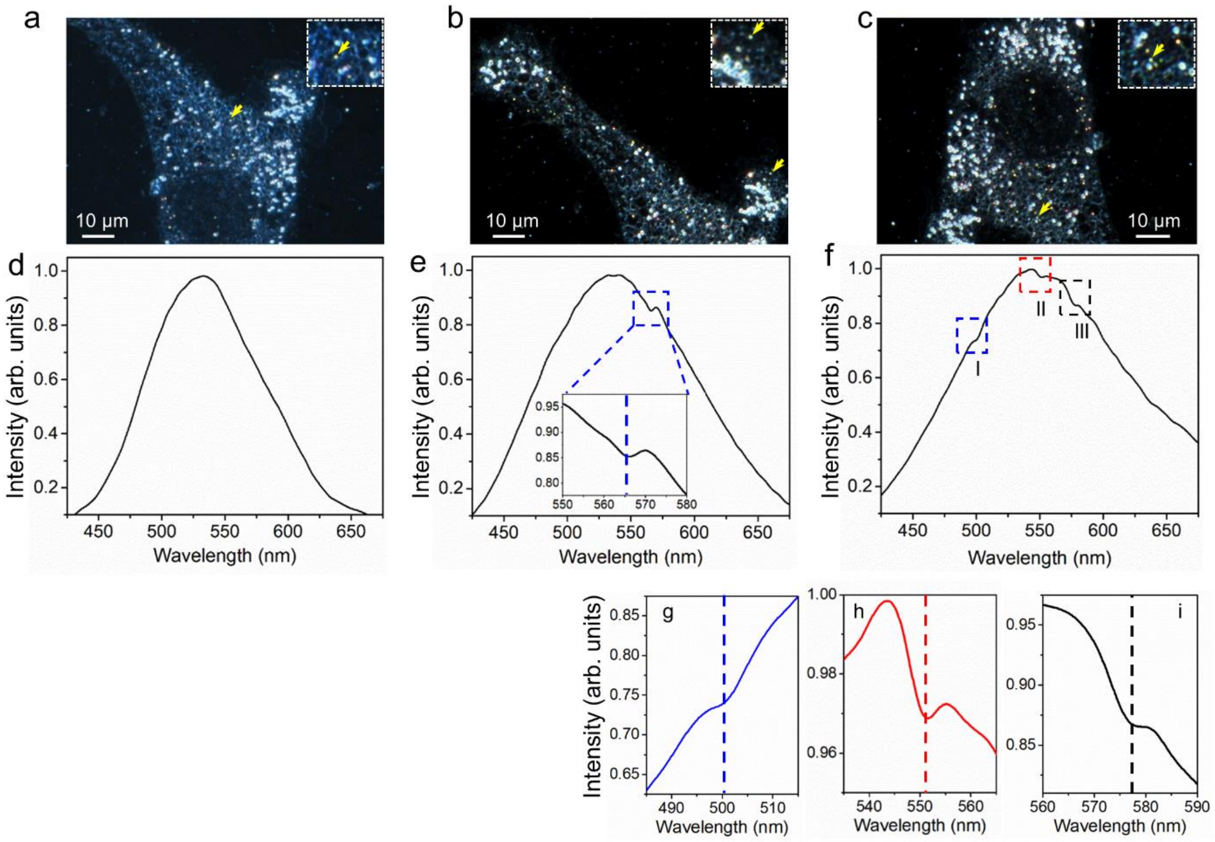

Supplementary Figure 18 | QBET detection with different dip signatures. (a)-(c) dark-field image of a cell with GNPs internalized, the yellow arrow indicates the GNP selected for the spectral analysis, inset shows enlarged image of the region with the target particle. (d)-(f) scattering spectra of GNPs indicated with yellow arrows in the upper panels. (a)&(d) No dip signature is detected. (b)&(e) Dip signature for other molecules rather than Cyt *c*, inset shows the dip at 565 nm. (c)&(f) Dip signatures for both Cyt *c* and other molecules, inset II indicates the dip for Cyt *c* (550 nm), while insets I and III indicate the dips for other molecules at resonant wavelengths of 500 and 575 nm, respectively. (g)-(i) shows the zoom-in image of inset I-III in (f), respectively.

Supplementary Table 1 | MTJ gap distance (*d*) with different linker molecules

a

$HS(CH_2)_nCOOH$

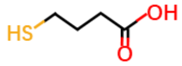

n	2	3	5	7	10
d (Å)	5.3	6.5	9.0	11.5	15.3

b

$HS(C_6H_4)_nCOOH$

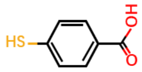

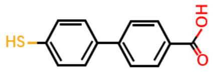

n	1	2
d (Å)	6.7	11.0

Supplementary Note 1 | Functional dynamics of Cyt *c* in mitochondria

In intact cells, Cyt *c* resides within the cristae of the inner mitochondrial membrane, and is functionally involved in ET within the electron transport chain. It transfers electrons between complexes III (Cyt *c* reductase) and IV (Cyt *c* oxidase, COX), which play a key role in the life-supporting function of ATP synthesis in mitochondrial as the powerhouse of the cell. With apoptotic stimulus, mitochondrial outer membrane permeabilisation is increased by the formation of pores or channels via different mechanisms such as oligomerisation of voltage-dependent anion channels (VDAC), B-cell lymphoma protein-2 (BCL2) effector proteins BAX and/or BAK, and VDAV-BAX. Under apoptotic stimulus, Cyt *c* is detached from inner mitochondrial membrane and released into the cytosol through these pores or channels on the outer mitochondrial membrane. Cyt *c* then serves as a key participant in cell death control through apoptosis activation.

Supplementary Note 2 | Discussion of electron tunnelling and transfer in the tunnel junction

The energy transfer from the GNP to Cyt *c* generated by electron tunnelling is governed by the eigenvalue in the Schrödinger equation: $\hat{H}\psi_i = E_i\psi_i$, where $\hat{H}$ is the Hamiltonian operator, and E_i is the quantized energy eigenvalue of the electron at the state of ψ_i. The electron at the state of ψ_i matching the electronic transition frequency of Cyt *c* is transferred from the GNP to Cyt *c*. Eventually, the scattering spectrum of the GNP with quantized dips at the electronic transition frequency of Cyt *c* is captured to image QBET in the tunnel junction. The probability $P(E_x)$ of an electron with energy of E_x to tunnel through a potential barrier $\varphi(x)$ in the x direction is given by the well-known WBK approximation[2], $P(E_x) = \exp\left\{-2\int_{x_1}^{x_2}\sqrt{[2m(V(x) - E_x)]/\hbar^2}\,dx\right\}$. For an estimated rectangular potential barrier with a tunnelling barrier width of d, the tunnelling probability of a single electron is then given by $P = \exp(-\beta d)$, where $\beta = 2K$ is the tunnelling decay rate with $K = \sqrt{2m(\varphi_0 - E)/\hbar^2}$, φ_0 is the potential barrier, E is the electron energy. By considering the cumulative effect of ET in the process and the excitation efficiency of Cyt *c* at different wavelengths, the ET efficiency is given by $Q = \alpha \exp(-\beta d)$, where α is a parameter describing the cumulative effect of the ET and Cyt *c* electronic excitation efficiency at a specific wavelength.

Supplementary Note 3 | Discussion on the conjugation between GNP and Cyt *c*

Cyt *c* has an isoelectric point in the range of 10.0-10.5, and carboxylic acid shows a selective binding to the functional binding sites on the front face of Cyt *c* which has positively charged residues[3]. Although conjugation of Cyt *c* to GNPs via some ligands can impact on the folding of the protein and in turn affects the redox state of the protein[4], the linker of carboxyl-terminated alkanethiol shows no significant changes in conformation and redox state of Cyt *c*[5]. This implies that conjugation of Cyt *c* to GNP via carboxyl-terminated linkers at neutral pH used in this study does not perturb the structure of Cyt *c* nor its electron transfer capability. There are four equivalent functional binding sites at the front face of Cyt c, *i.e.*, Gly^{1}-Phe^{10}, Ile^{95}-Glu^{104}, Tyr^{67}-Met^{80}, and Tyr^{67}-Phe^{82}. The final conjugation between the linker molecule and Cyt *c* is determined by the balance between electrostatics and hydrophobicity, with a binding energy of about 9.3-10.0 kcal/mol. Conjugation of linker molecules with different binding sites on Cyt *c* may lead to slight variations in the tunnelling distance and potentially results in a heterogeneity of spectra response. Therefore, the final spectra response at the steady-state of conjugation is an overall average encompassing Cyt *c* bound at different sites.

Supplementary Note 4 | Description of Cyt *c* reduction in bulk solution

The reaction process in bulk solution was captured via real-time absorption peak changes from 530 nm to 520 and 550 nm. At the same concentration of reductant (10 mM), the reduction took more than 2 h. To quantitatively compare the two reactions, we normalised the real-time spectra intensity change at 530 and 550 nm to represent the normalised concentration of Cyt *c* (Ox.) and Cyt *c* (Red.), respectively (Fig. 3f & 3g). In both reactions, the concentration of $S_2O_4^{2-}$ is much higher than that of Cyt *c*, thus we assumed the concentration of $S_2O_4^{2-}$ is constant during the reaction. Based on this assumption, the pseudo-first-order kinetics with respect to Cyt *c* (Ox.) can be applied to the reaction[6] which is given by $[\text{Cyt } c \text{ (Ox.)}] = [\text{Cyt } c \text{ (Ox.)}]_0 \exp(-kt)$, where $[\text{Cyt } c \text{ (Ox.)}]$ and $[\text{Cyt } c \text{ (Ox.)}]_0$ are the real-time and initial concentration of Cyt *c* (Ox.), respectively, and k is the reaction rate constant. The good linear correlation of ln [Cyt *c* (Ox.)] with time (Fig. 3h) further supports the pseudo-first-order assumption.

Supplementary Note 5 | Discussion of plasmonic peak shift and QBET detection

It is noted that some of the particles form into aggregates with a redshift in plasmonic resonance peak (λ_{max}) during the capture of QBET in mitochondria extracts. Although λ_{max} of the aggregates were redshifted than that of the single particle, the quantized dips were still detectable in the scattering spectra if the scattering spectra show an overlap with absorption peaks of Cyt *c* (Supplementary Fig. 17b). However, the dip depth becomes smaller as λ_{max} gradually shifts away from the molecular resonance peak. This is probably due to the attenuated electron transfer by tunnelling. Particularly when λ_{max} drifts away from the absorption peak (for example, $\lambda_{max} > 600$ nm), the scattering intensity at the wavelength of Cyt *c* absorption peak detected was smaller than 30% of that at λ_{max}. Under such conditions, QBET was not captured due to the absence of quantized dips.

Supplementary Note 6 | Curve fit description

In Fig. 4d and 4 h in the main text, the smooth curve is generated using a cubic B-spline connection of the experimental data, and this connection is directly generated from the data analysis software OriginLab. The B-spline curve around each data point ($X[i]$, $Y[i]$), i = 1, 2, … n, can be described by parametric equations, as shown below:

$$X_i[t] = \frac{1}{6}\{(-t^3 + 3t^2 - 3t + 1)\,X[i-1] + (3t^3 - 6t^2 + 3t)\,X[i] + (-3t^3 + 3t^2)\,X[i+1] + t^3\,X[i+2]\};$$

$$Y_i[t] = \frac{1}{6}\{(-t^3 + 3t^2 - 3t + 1)\,Y[i-1] + (3t^3 - 6t^2 + 3t)\,Y[i] + (-3t^3 + 3t^2)\,Y[i+1] + t^3\,Y[i+2]\},$$

where $2 \leqslant i \leqslant n$-2, and $0 \leqslant t \leqslant 1$.

Supplementary References

1. Hansen, T. *et al.* Orbital Topology Controlling charge injection in quantum-dot-sensitized solar cells. *J. Phys. Chem. Lett.* **5**, 1157-1162 (2014).
2. Simmons, J. G. Generalized formula for the electric tunnel effect between similar electrodes separated by a thin insulating film. *J. Appl. Phys.* **34**, 1793-1803 (1963).
3. Bayraktar, H., You, C.-C., Rotello, V. M. & Knapp, M. J. Facial control of nanoparticle binding to cytochrome c. *J. Am. Chem. Soc.* **129**, 2732-2733 (2007).
4. Aubin-Tam, M.-E. & Hamad-Schifferli, K. Gold nanoparticle-cytochrome C complexes: the effect of nanoparticle ligand charge on protein structure. *Langmuir* **21**, 12080-12084 (2005).
5. Chen, X., Ferrigno, R., Yang, J. & Whitesides, G. M. Redox properties of cytochrome c adsorbed on self-assembled monolayers: a probe for protein conformation and orientation. *Langmuir* **18**, 7009-7015 (2002).
6. Zeng, J., Zhang, Q., Chen, J. & Xia, Y. A comparison study of the catalytic properties of Au-based nanocages, nanoboxes, and nanoparticles. *Nano Lett.* **10**, 30-35 (2010).
