# Supplementary material for: Quantum biological tunnel junction for electron transfer imaging in live cells
Source: Nat Commun. 2019 Jul 19;10:3245. doi: 10.1038/s41467-019-11212-x (PMC6642182; doi:10.1038/s41467-019-11212-x)
Supplement: Supplementary file 2 — Description of Additional Supplementary Files [file 41467_2019_11212_MOESM2_ESM.pdf]

Description of Additional Supplementary Files

File Name: Supplementary Movie 1

Description: HeLa cell with GNP uptake. Organelles and GNPs inside the cell as well as their movement can be clearly seen.
